# Different dietary restriction regimens extend lifespan by both independent and overlapping genetic pathways in *C. elegans*

**DOI:** 10.1111/j.1474-9726.2009.00459.x

**Published:** 2009-04

**Authors:** Eric L Greer, Anne Brunet

**Affiliations:** 1 Department of GeneticsStanford CA 94305, USA; 2 Cancer Biology ProgramStanford CA 94305, USA; 3 Neurosciences Program, 300 Pasteur Drive, Stanford UniversityStanford CA 94305, USA

**Keywords:** aging, AMPK, dietary restriction, FoxO transcription factors, longevity, resveratrol

## Abstract

Dietary restriction (DR) has the remarkable ability to extend lifespan and healthspan. A variety of DR regimens have been described in species ranging from yeast to mammals. However, whether different DR regimens extend lifespan via universal, distinct, or overlapping pathways is still an open question. Here we examine the genetic pathways that mediate longevity by different DR regimens in *Caenorhabditis elegans*. We have previously shown that the low-energy sensing AMP-activated protein kinase AMPK/*aak-2* and the Forkhead transcription factor FoxO/*daf-16* are necessary for longevity induced by a DR regimen that we developed (sDR). Here we find that AMPK and FoxO are necessary for longevity induced by another DR regimen, but are dispensable for the lifespan extension induced by two different DR methods. Intriguingly, AMPK is also necessary for the lifespan extension elicited by resveratrol, a natural polyphenol that mimics some aspects of DR. Conversely, we test if genes previously reported to mediate longevity by a variety of DR methods are necessary for sDR-induced longevity. Although *clk-1*, a gene involved in ubiquinone biosynthesis, is also required for sDR-induced lifespan extension, we find that four other genes (*sir-2.1*, FoxA/*pha-4*, *skn-1*, and *hsf-1*) are all dispensable for longevity induced by sDR. Consistent with the observation that different DR methods extend lifespan by mostly independent genetic mechanisms, we find that the effects on lifespan of two different DR regimens are additive. Understanding the genetic network by which different DR regimens extend lifespan has important implications for harnessing the full benefits of DR on lifespan and healthspan.

## Introduction

Restricting nutrients without malnutrition extends lifespan and reduces age-dependent decline and diseases in virtually all species (Masoro, 2005). Although a single term [dietary restriction (DR)] is often used to refer to this intervention, there exist a number of methods of restricting nutrients that all result in lifespan extension in species ranging from yeast to mice (Goodrick *et al*., 1990; Mair *et al*., 2005; Masoro, 2005; Dilova *et al*., 2007; Mair & Dillin, 2008; Piper & Bartke, 2008; Skorupa *et al*., 2008). Whether the different methods of restricting nutrients converge on a common pathway to extend lifespan or whether independent mechanisms are elicited depending on how DR is achieved is still unclear.

The nematode *Caenorhabditis elegans* provides a good model to study the genetics of lifespan in response to different DR regimens. There exist eight distinct methods of manipulating the diet that all extend lifespan to varying degrees in *C. elegans*, allowing comparison across DR regimens (Table 1). The standard worm diet consists of attenuated *E. coli* bacteria (OP50) placed on agarose plates. The DR methods in worms are: (i) a genetic mutation (*eat-2*) that reduces the pharyngeal pumping rate of the worms, thereby decreasing food intake (Avery, 1993; Lakowski & Hekimi, 1998); (ii and iii) two different methods of diluting the bacteria in liquid cultures (bacterial DR: bDR and liquid DR: lDR) (Klass, 1977; Houthoofd *et al*., 2003; Bishop & Guarente, 2007; Panowski *et al*., 2007); (iv and v) two chemically defined liquid medias that induce DR-like phenotype in *C. elegans* (axenic medium and chemically defined liquid medium: CDLM) (Houthoofd *et al*., 2002a; Szewczyk *et al*., 2006); (vi) the dilution of peptone in the agarose plates, which reduces the growth of bacteria (DP: dilution of peptone) (Hosono *et al*., 1989); (vii) the total absence of bacteria on plates (dietary deprivation: DD) (Kaeberlein *et al*., 2006; Lee *et al*., 2006); and (viii) a method that we recently described where bacteria are serially diluted on plates (solid DR: sDR) (Greer *et al*., 2007).

**Table 1:** Eight methods of dietary restriction in *C. elegans*

Method	Normal conditions	*eat-2*	bDR	lDR	Axenic	CDLM	DP	DD	sDR	Resveratrol
Medium	Solid	Solid	Liquid	Liquid + Solid	Liquid	Liquid	Solid	Solid	Solid	Solid
Source of food	Live *E. coli*	Live *E. coli*	Live *E. coli* (antibiotics)	Live *E. coli* (antibiotics)	Defined chemical broth	Defined chemical broth	Live *E. coli*	No *E. coli*	Live or dead *E. coli*†	Live or dead *E. coli*†
Genetic mutation		Mutation in the non-α-nicotinic acetylcholine receptor subunit	No	No	No	No	No	No	No	No
Temporal		Birth	Day 2 of adulthood	L4/young adult	Larval day 4 (L4)	Birth	Birth	Day 2 of adulthood	Day 4 of adulthood	Birth
Percentage of Lifespan extension* (%)		0–57	60–73	28	80–150	88	33	42–50	18–35	6–14
Effect on fertility		Decrease	Decrease	Decrease	Decrease	Decrease	Increase	ND	Decrease‡	No effect
Used by		[1–6]	[2,7,18]	[8]	[6,7,9]	[10]	[11]	[3,4,12]	[13]	[14–17]

* Independent laboratories calculate lifespan starting at different ages (birth vs. young adult).

† While UV-killed bacteria were used in some experiments with sDR and resveratrol, most experiments were performed with live bacteria.

‡ sDR is normally initiated at a post-reproductive age (day 4 of adulthood), but when initiated at day 1 of adulthood, sDR decreases fertility (data not shown).

ND, not determined.

[1]: (Lakowski & Hekimi, 1998), [2]: (Panowski *et al*., 2007), [3]: (Kaeberlein *et al*., 2006), [4]: (Lee *et al*., 2006), [5]: (Hansen *et al*., 2007), [6]: (Houthoofd *et al*., 2002b), [7]: (Houthoofd *et al*., 2003), [8]: (Bishop & Guarente, 2007), [9]: (Houthoofd *et al*., 2002a), [10]: (Szewczyk *et al*., 2006), [11]: (Hosono *et al*., 1989), [12]: (Steinkraus *et al*., 2008), [13]: (Greer *et al*., 2007), [14]: (Wood *et al*., 2004), [15]: (Viswanathan *et al*., 2005), [16]: (Bass *et al*., 2007), [17]: (Gruber *et al*., 2007), [18]: (Klass, 1977).

In addition to methods that restrict the diet, a number of chemical compounds have been proposed to act as ‘DR mimetics’, which extend lifespan without inducing the detrimental effects of restricting food (Ingram *et al*., 2006). For example, the natural polyphenol compound resveratrol has been proposed to act as a DR mimetic in yeast (Howitz *et al*., 2003), worms (Wood *et al*., 2004; Viswanathan *et al*., 2005; Gruber *et al*., 2007), flies (Wood *et al*., 2004), fish (Valenzano *et al*., 2006), and mice on a high fat diet (Baur *et al*., 2006), although resveratrol did not extend lifespan in flies in one study (Bass *et al*., 2007) and in mice on a normal diet (Pearson *et al*., 2008). While resveratrol and various DR regimens can significantly extend lifespan, whether they do so by universal, independent, or overlapping mechanisms is unknown.

A number of genes mediating longevity by different DR methods or by resveratrol have recently been uncovered in invertebrates (Lakowski & Hekimi, 1998; Wood *et al*., 2004; Hansen *et al*., 2005; Viswanathan *et al*., 2005; Wang & Tissenbaum, 2006; Bishop & Guarente, 2007; Greer *et al*., 2007; Hansen *et al*., 2007; Panowski *et al*., 2007; Steinkraus *et al*., 2008). The NAD-dependent deacetylase of the Sir2 family was one of the first genes identified to be necessary for longevity induced by various DR regimens and by resveratrol in yeast, worms, flies, and possibly mice (Lin *et al*., 2000; Rogina & Helfand, 2004; Wood *et al*., 2004; Chen *et al*., 2005; Viswanathan *et al*., 2005; Wang & Tissenbaum, 2006), although the importance of Sir2 in DR- or resveratrol-induced longevity is not always observed in these organisms (Kaeberlein *et al*., 2004; Kaeberlein *et al*., 2006; Lee *et al*., 2006; Bass *et al*., 2007; Hansen *et al*., 2007). Another pathway that was identified in yeast, worms, and flies, to mediate DR induced longevity is the amino-acid sensing TOR pathway (Kapahi *et al*., 2004; Kaeberlein *et al*., 2005; Hansen *et al*., 2007), although TOR is not always necessary for DR induced longevity in *C. elegans* (Henderson *et al*., 2006). In addition, a series of transcriptional regulators involved in the response to oxidative stress have recently been implicated in DR in *C. elegans*: the Forkhead transcription factor FoxA/*pha-4* is necessary for longevity induced by the *eat-2* mutation and by bDR (Panowski *et al*., 2007); the Nrf2 transcription factor *skn-1* is necessary for lifespan extension by lDR (Bishop & Guarente, 2007); and the heat-shock transcription factor *hsf-1* plays an important role in longevity triggered by DD (Steinkraus *et al*., 2008), though *hsf-1* is dispensable for *eat-2* induced lifespan extension (Hsu *et al*., 2003). Finally, *clk-1*, a gene encoding a mitochondrial protein involved in ubiquinone synthesis, also appears to be required for longevity induced by the *eat-2* mutation in worms (Lakowski & Hekimi, 1998).

We have recently discovered that the low energy-sensing kinase AMPK/*aak-2* is necessary for longevity induced by sDR in worms (Greer *et al*., 2007). AMPK can act upstream of the Forkhead transcription factor FoxO/*daf-16* to extend lifespan, perhaps via direct phosphorylation (Greer *et al*., 2007). Like AMPK, FoxO is necessary for longevity induced by sDR (Greer *et al*., 2007). In contrast, neither AMPK nor FoxO are necessary for the longevity induced by *eat-2* (Lakowski & Hekimi, 1998; Curtis *et al*., 2006). In addition, FoxO is not necessary for longevity induced by other DR methods (bDR, lDR, axenic medium, and DD) (Houthoofd *et al*., 2003; Kaeberlein *et al*., 2006; Lee *et al*., 2006; Bishop & Guarente, 2007; Panowski *et al*., 2007). Similarly, in Drosophila, FoxO is not absolutely necessary for DR-induced longevity (Giannakou *et al*., 2008; Min *et al*., 2008), although FoxO alters the optimal food concentration required for longevity (Clancy *et al*., 2002; Giannakou *et al*., 2008; Min *et al*., 2008). In mammals, the role of AMPK and FoxO in DR-induced longevity has not been examined yet. While a series of genes have been identified as playing important roles in longevity induced by different DR methods, the comparison of the importance of these genes in diverse DR regimens has not been performed. Identifying the different genetic pathways by which the various methods of restricting nutrients promote longevity is important for harnessing the full benefits of DR on lifespan.

Here we test whether distinct DR methods are mediated by specific or common genetic pathways. We find that while AMPK and FoxO are necessary for longevity induced by sDR and by peptone dilution in plates, these genes are not absolutely required for *eat-2* and bDR to extend lifespan. Intriguingly, AMPK, but not FoxO, is necessary for the DR mimetic resveratrol to extend lifespan in worms. We then test whether sDR is mediated by genes that were previously found to mediate longevity by other DR methods or DR mimetics. We find that *sir-2.1*, *pha-4, skn-1*, and *hsf-1* are all dispensable for sDR-induced lifespan extension, but that *clk-1* is necessary for this regimen to extend lifespan. Finally, we show that sDR further enhances the lifespan of *eat-2* mutant worms, indicating that these two DR methods act additively to promote longevity. Our results are compatible with a model in which sDR induces lifespan extension by a mechanism that is different from, but overlapping with, that of other DR methods. Understanding how different methods of DR induce lifespan extension is pivotal for the identification of all the components of the gene network that orchestrates maximal longevity extension in response to nutrient deprivation.

## Results

### AMPK/*aak-2* and FoxO/*daf-16* are necessary for sDR-induced lifespan extension across a gradient of bacteria on plates

**Fig. 1:**
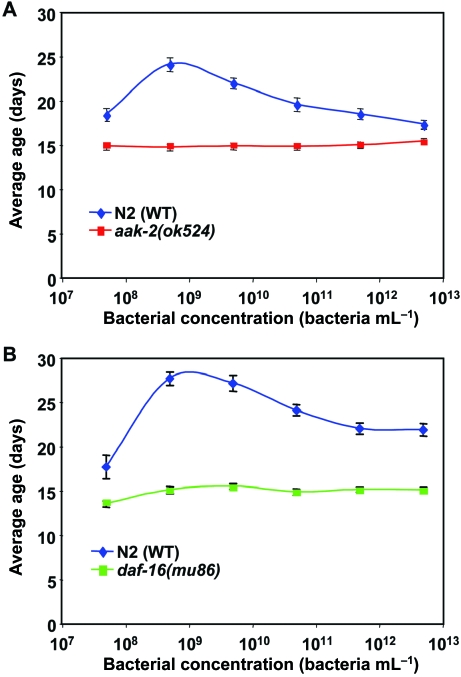
AMPK/*aak-2* and FoxO/*daf-16* are necessary for lifespan extension by sDR across a gradient of bacteria. (A) A serial dilution of bacteria on plates (5 × 10^12^ to 5 × 10^7^ bacteria mL^−1^) extends WT (N2) worm lifespan but does not extend *aak-2(ok524)* mutant worm lifespan. Two-way anova revealed that the lifespan extension of WT (N2) worms across a bacterial gradient was significantly different from that of *aak-2(ok524)* mutant worms (*P* < 0.0001). Mean, standard errors, and statistical analysis for two independent experiments performed in triplicate are presented in Table S1A. (B) A serial dilution of bacteria on plates extends WT (N2) worm lifespan but does not extend *daf-16(mu86)* mutant worm lifespan. Two-way anova revealed that the lifespan extension of WT (N2) worms across a bacterial gradient was significantly different from that of *daf-16 (mu86)* mutant worms (*P* < 0.0001). Mean, standard errors, and statistical analysis for one experiment performed in triplicate are presented in Table S1B.

Assessing lifespan at only two concentrations of food (*ad libitum* vs. DR) does not distinguish genes that are truly involved in mediating DR from genes that affect the optimal response to food concentrations required to elicit the DR effect (e.g. *chico* in flies) (Clancy *et al*., 2002). Therefore, to test if AMPK/*aak-2* and FoxO/*daf-16* mediated lifespan extension in response to DR or affected the optimal response to food concentration, we performed lifespan experiments using a gradient of bacterial concentrations. The lifespan extension of WT worms as a function of bacteria concentration follows a parabola-shaped curve (Fig. 1), with moderate reduction of food intake leading to beneficial effects on lifespan (DR) and severe reduction of food intake having detrimental effects leading to death (starvation) (Piper & Partridge, 2007; Mair & Dillin, 2008). In contrast, the lifespan of worms carrying a null mutation in the *aak-*2 or in the *daf-16* gene (*aak-2*(*ok524*) *and daf-16*(*mu86*)) was never extended, regardless of the concentration of bacteria (Fig. 1A,B; Tables S1A and S1B in Supporting Information). These results indicate that AMPK/*aak-2* and FoxO/*daf-16* are necessary for lifespan extension in response to sDR and that mutations in these genes do not lead to a shift of the overall organismal dependence on food.

**Fig. 5:**
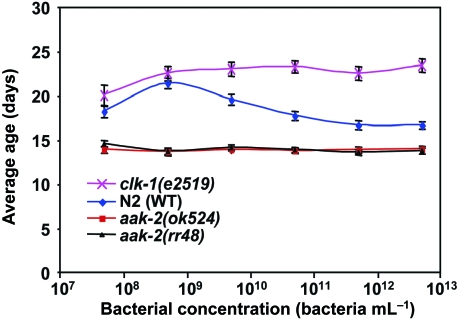
*clk-1* is necessary for sDR to extend lifespan. A serial dilution of bacteria on plates extended WT (N2) worm lifespan (28.5%, *P* < 0.0001) but did not extend two independent *aak-2* mutant strains, *aak-2*(*rr48*) (1.0%*P* = 0.5787) and *aak-2*(*ok524*) (–1.4%*P* = 0.7804), or *clk-1*(*e2519*) mutant worm lifespans (0%*P* = 0.6921). Two-way anova revealed that the lifespan extension of WT (N2) worms across a bacterial gradient was significantly different from that of *aak-2*(*ok524*) mutant worms (*P* < 0.0001), *aak-2*(*rr48*) mutant worms (*P* < 0.0001), or *clk-1*(*e2519*) mutant worms (*P* < 0.0001). Mean, standard errors, and statistical analysis for two independent experiments performed in triplicate are presented in Table S9.

The dependency of sDR on AMPK and FoxO was also observed with independent backcrossed worm strains (*aak-2*(*rr48*) for AMPK and *daf-16(m26)* or *daf-16*(*mgDf50)* for FoxO) (Fig. 5, data not shown), with bacterial strains from independent laboratories (data not shown), and in the presence or absence of FUdR, an inhibitor of worm reproduction (Figure S1; Table S1C). Together with our previous findings (Greer *et al*., 2007), these results indicate that AMPK and FoxO are necessary to mediate longevity induced by sDR, a regimen that restricts food intake in worms.

### AMPK and FoxO are necessary for the lifespan extension due to peptone dilution

**Fig. 2:**
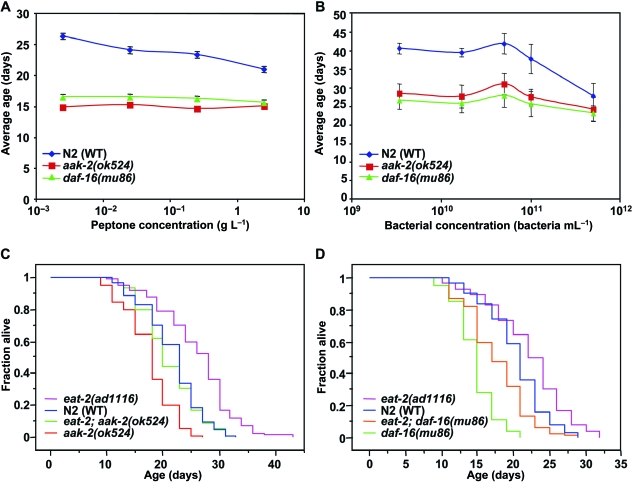
AMPK and FoxO mediate longevity induced by some but not all DR methods. (A) Longevity induced by dilution of peptone (DP) is dependent on AMPK/*aak-2* and FoxO/*daf-16*. Dilution of peptone in the plates extends WT (N2) worm lifespan (25.4%, *P* < 0.0001) but does not extend *aak-2*(*ok524*) mutant lifespan (–1.0%, *P* = 0.6371) or *daf-16*(*mu86*) mutant lifespan (5.7%, *P* = 0.1402). Two-way anova revealed that the lifespan extension of WT (N2) worms across a peptone gradient was significantly different from that of *aak-2*(*ok524*) mutant worms (*P* < 0.0001) or *daf-16(mu86)* mutant worms (*P* < 0.0001). Mean, standard errors, and statistical analysis for two independent experiments performed in triplicate are presented in Table S2. (B) AMPK/*aak-2* and FoxO/*daf-16* are not completely necessary for bDR lifespan extension. The average and SEM of three independent experiments indicates that bDR increases WT (N2), *aak-2*(*ok524*), and *daf-16*(*mu86*) mutant lifespan but appears to increase WT (N2) worm lifespan to a greater extent than *aak-2*(*ok524*) or *daf-16*(*mu86*) mutant worm lifespans. For each individual experiments, see Figure S2 and Table S3. (C) As previously reported (Curtis *et al*., 2006), the *eat-2*(*ad1116*) mutation extends WT (N2) worm lifespan (19.8%, *P* < 0.0001) and *aak-2*(*ok524*) mutant lifespan (19.4%, *P* < 0.0001). Mean, standard errors, and statistical analysis for two independent experiments performed in triplicate are presented in Table S4A. (D) As previously reported (Lakowski & Hekimi, 1998), the *eat-2*(*ad1116*) mutation extends WT (N2) worm lifespan (12.9%, *P* < 0.0001) and *daf-16*(*mu86*) mutant lifespan (32.3%*P* < 0.0001). Mean, standard errors, and statistical analysis for two independent experiments performed in triplicate are presented in Table S4B.

We next asked how general the role of AMPK and FoxO was in longevity induced by different methods of restricting nutrients. Dilution of peptone in bacterial plates extends worm lifespan because it is considered to reduce bacterial growth on the plates (Hosono *et al*., 1989). We find that the dilution of peptone in bacterial plates extends lifespan in an AMPK/*aak-2* and FoxO/*daf-16* dependent manner (*P* < 0.0001 by two-way anova) (Fig. 2A; Table S2). This result indicates that AMPK and FoxO are also necessary for lifespan extension induced by another method of restricting nutrients.

### AMPK and FoxO are not completely necessary for bDR and *eat-2* induced longevity

FoxO/*daf-16* was shown not to be entirely necessary for longevity induced by the dilution of bacteria in liquid cultures (bDR) (Houthoofd *et al*., 2003; Panowski *et al*., 2007). However, the importance of AMPK in bDR-induced lifespan extension has never been examined. We tested whether AMPK and FoxO were required for lifespan extension in response to a gradient of bacteria concentrations in liquid culture. Combining the results of three independent experiments (Fig. 2B), we found that bDR significantly extended the lifespan of WT (N2), *aak-2*(*ok524*), and *daf-16*(*mu86*) mutant worms (*P* < 0.0001), indicating that AMPK and FoxO are dispensable for the entire lifespan extension induced by bDR. In two of these experiments, bDR extended WT (N2) lifespan to a larger extent than *aak-2* and *daf-16* mutant worm lifespan (*P* < 0.0001 by two-way anova) (Figure S2A and S2B; Table S3). In one experiment however, bDR extended the lifespan of WT (N2) worms to a similar extent as that of *aak-2* and *daf-16* mutant worms (*P* = 0.1528 and 0.6643 respectively by two-way anova) (Figure S2C; Table S3). Note that in one of the assays (Figure S2A), starvation was not reached, which may alter the interpretation of the experiment. However, when this assay was omitted for statistical analysis, we still found that bDR extended WT (N2) lifespan to a larger extent than *aak-2* and *daf-16* mutant worm lifespan (*P* < 0.001 by two-way anova). Although the parameters for these variations are unknown, these results suggest that AMPK and FoxO are dispensable for bDR to extend lifespan, but that they play a modulatory role in the extension of lifespan by this regimen.

Finally, as previously reported, we confirmed that AMPK/*aak-2* and FoxO/*daf-16* were completely dispensable for the lifespan extension of *eat-2*(*ad1116*) mutant worms, a genetic way to mimic DR (Lakowski & Hekimi, 1998; Curtis *et al*., 2006) (Fig. 2C,D; Table S4). Together, these results indicate that AMPK and FoxO are required for longevity induced by some (sDR and DP) but not all (bDR and *eat-2*) DR methods.

### Resveratrol extends lifespan in an AMPK-dependent, but FoxO-independent, manner

**Fig. 3:**
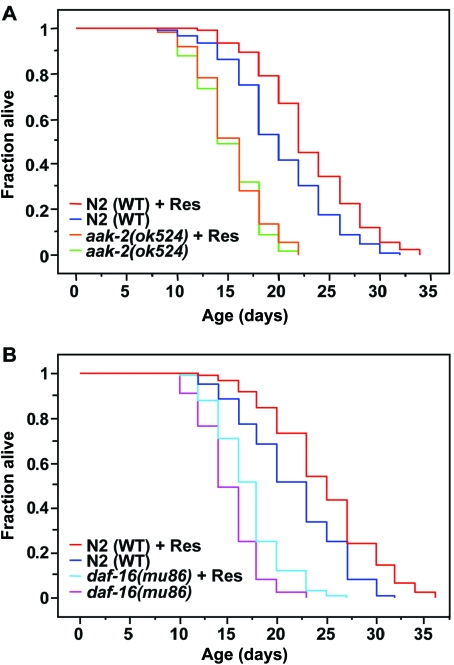
Resveratrol extends lifespan in an AMPK-dependent, but FoxO-independent, manner. (A) Resveratrol (100 µm) extended WT (N2) worm lifespan (14.2%, *P* = 0.0005), but did not significantly extend *aak-2*(*ok524*) mutant worm lifespan (2.2%*P* = 0.5485). Mean, standard errors, and statistical analysis for three independent experiments performed in triplicate are presented in Table S5. (B) Resveratrol (100 µm) extended both WT (N2) worm lifespan (14.6%*P* < 0.0001) and *daf-16*(*mu86*) mutant worm lifespan (13.7%*P* < 0.0001). Res, resveratrol. Mean, standard errors, and statistical analysis for one experiment performed in triplicate are presented in Table S5.

Resveratrol extends lifespan in many species and has been suggested to act as a DR mimetic (Sinclair, 2005). Resveratrol has recently been found to activate AMPK in cultured cells and in mice (Baur *et al*., 2006; Zang *et al*., 2006; Dasgupta & Milbrandt, 2007; Hwang *et al*., 2007). We thus asked whether AMPK was necessary for increased longevity induced by resveratrol in *C. elegans*. Resveratrol induced a modest, but statistically significant, increase in WT (N2) worm lifespan. In contrast, resveratrol did not increase the lifespan of *aak-2*(*ok524*) mutant worms, suggesting that AMPK is necessary for the beneficial effects of resveratrol on lifespan (Fig. 3A; Table S5), as was proposed by (Bass *et al*., 2007). Similar to what was previously reported (Viswanathan *et al*., 2005), we found that resveratrol still extended the lifespan of *daf-16*(*mu86*) mutant worms (Fig. 3B; Table S5), indicating that resveratrol extends lifespan by eliciting an AMPK-dependent, FoxO-independent pathway. Therefore, activation of AMPK is not always coupled to that of FoxO, even though AMPK's ability to extend lifespan is dependent on the presence of FoxO (Greer *et al*., 2007). This result suggests that AMPK also regulates other substrates to extend lifespan, which is consistent with published findings (Apfeld *et al*., 2004; Narbonne & Roy, 2006). Therefore, these results suggest that different ways of manipulating nutrients and chemical compounds extend lifespan via independent and non-linear genetic pathways.

### *sir-2.1*, *pha-4*, *skn-1*, and *hsf-1* are not necessary for sDR-induced lifespan extension

Having shown that FoxO and AMPK were important for some, but not all, DR methods, we next tested if conversely, genes that have been implicated in lifespan extension by resveratrol or by other DR regimens were necessary for sDR to extend lifespan.

**Fig. 4:**
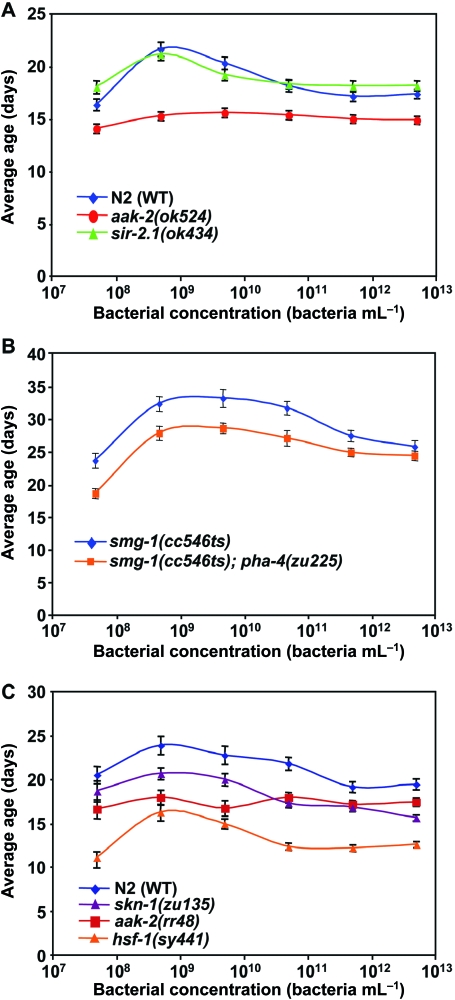
*sir-2.1*, F*oxA/pha-4*, *skn-1* and *hsf-1* are not entirely necessary for sDR to extend lifespan. (A) A serial dilution of bacteria on plates (5 × 10^12^ to 5 × 10^7^ bacteria mL^−1^) extends WT (N2) (26.1%, *P* < 0.0001) and *sir-2.1*(*ok434*) (16.6%, *P* < 0.0001) mutant worm lifespan but does not extend *aak-2*(*ok524*) (1.3%, *P* = 0.6330) mutant worm lifespan. Two-way anova revealed that the lifespan extension of WT (N2) worms across a bacterial gradient was significantly different from that of *aak-2*(*ok524*) mutant worms (*P* < 0.0001) but not statistically different from *sir-2.1*(*ok434*) mutant worms (*P* = 0.1240). Mean, standard errors, and statistical analysis for three independent experiments performed in triplicate are presented in Table S6. (B) A serial dilution of bacteria on plates extended *smg-1*(*cc546ts*) and *smg-1*(*cc546ts*); *pha-4*(*zu225*) mutant worms to a similar extend (*P* = 0.3724 by two-way anova). Note that this experiment was performed at 15 °C after worms reached adulthood. Mean, standard errors, and statistical analysis for two independent experiments performed in triplicate are presented in Table S7A. (C) A serial dilution of bacteria on plates extended WT (N2) (24.9%, *P* < 0.0001) worm lifespan, *skn-1*(*zu135*) (23.1%, *P* < 0.0001) mutant worm lifespan, and *hsf-1*(*sy441*) (33.5%, *P* < 0.0001) mutant worm lifespan but did not extend *aak-2*(*rr48*) (4.6%, *P* = 0.2566) mutant worm lifespan. Two-way anova revealed that the lifespan extension of WT (N2) worms across a bacterial gradient was significantly different from that of *aak-2*(*ok524*) mutant worms (*P* < 0.0001) but not statistically different from *skn-1*(*zu135*) mutant worm lifespan (*P* = 0.5567) or *hsf-1*(*sy441*) mutant worm lifespan (*P* = 0.2843). Mean, standard errors, and statistical analysis for two independent experiments performed in triplicate are presented in Table S8 and Table S9.

We first asked if *sir-2.1,* which encodes a Sirtuin family protein deacetylase, was required for lifespan extension in response to sDR. *sir-2.1 has* been found to mediate lifespan extension by resveratrol in some studies (Wood *et al*., 2004; Viswanathan *et al*., 2005), but not others (Bass *et al*., 2007). We used a mutant strain carrying a deletion in the *sir-2.1* gene, which is predicted to be a null mutant (*sir-2.1*(*ok434*)) (Wang & Tissenbaum, 2006). We found that sDR increased the lifespan of both WT and *sir-2.1* mutant worms to the same extent (Fig. 4A; Table S6). There was no statistically significant difference between the effects of sDR on the longevity of WT worms vs. *sir-2.1* mutant worms (*P* = 0.1240 by two-way anova). These results indicate that *sir-2.1* is not necessary for lifespan extension by sDR.

We then tested the importance of the Forkhead transcription factor FoxA/*pha-4*, a gene involved in *eat-2* and bDR-induced longevity (Panowski *et al*., 2007), in lifespan extension induced by sDR. We used a temperature sensitive allele of FoxA/*pha-4*, *smg-1*(*cc546ts*); *pha-4*(*zu225*), and its control counterpart *smg-1*(*cc546ts*) (Gaudet & Mango, 2002). When shifted to the restrictive temperature *smg-1*(*cc546ts*); *pha-4*(*zu225*) display very little PHA-4 protein and the phenotype of *smg-1*(*cc546ts*); *pha-4*(*zu225*) is similar to that of the *pha-4*(*q490*) null mutant (Kaltenbach *et al*., 2005; Kiefer *et al*., 2007). We found that sDR extended lifespan of both the control strain (*smg-1*(*cc546ts*)) and the inducible *pha-4* mutant strain (*smg-1*(*cc546ts*)*; pha-4*(*zu225*)) (Fig. 4B; Table S7A) (*P* = 0.3724 by two-way anova). These results indicate that FoxA/*pha-4* is not necessary for sDR induced lifespan extension. Consistent with this observation, we found that sDR still increased the lifespan of worms in which FoxA/*pha-4* was knocked-down by RNAi, but did not extend the lifespan of worms in which FoxO/*daf-16* was knocked-down (Figure S3; Table S7B). These findings suggest that FoxO, but not FoxA, is required for sDR-induced longevity.

We also examined if *skn-1*, a gene encoding a transcription factor necessary for lDR to extend lifespan (Bishop & Guarente, 2007), was required for longevity in response to sDR. We used a loss of function mutant strain of *skn-1*, *skn-1*(*zu135*), which displays a premature stop codon in all three isoforms of SKN-1 (a, b, and c) (Bishop & Guarente, 2007; Tullet *et al*., 2008). We showed that sDR extended lifespan of WT worms and *skn-1*(*zu135*) mutant worms to a similar extent (*P* = 0.5567 by two-way anova). Although it is formally possible that some SKN-1 activity remains in the *skn-1*(*zu135*) mutant strain, these findings nevertheless suggest that *skn-1* is not necessary for sDR-induced longevity (Fig. 4C; Table S8).

Finally, we asked if *hsf-1*, which encodes a heat-shock responsive transcription factor involved in longevity in response to DD (Steinkraus *et al*., 2008), was necessary for sDR-induced lifespan extension. We used a mutant strain of *hsf-1* (*hsf-1*(*sy441*)) that contains a premature stop codon that eliminates the transactivation domain of HSF-1 and is likely to be a null mutant (Hajdu-Cronin *et al*., 2004). We found that sDR still extended the lifespan in *hsf-1*(*sy441*) mutant worms similarly to WT worms (*P* = 0.2843 by two-way anova), indicating that *hsf-1* is not necessary for sDR-induced longevity (Fig. 4C; Table S9).

Together, these data indicate that four genes (*sir-2.1*, *pha-4*, *skn-1*, and *hsf-1*) that have been previously implicated in longevity in response to a variety of DR methods and DR mimetics do not mediate lifespan extension by sDR. These findings further corroborate the observation that different DR regimens evoke independent pathways.

### *clk-1* is necessary for sDR-induced lifespan extension

The *clk-1* gene encodes a demethoxyubiquinone hydroxylase that is necessary for the biosynthesis of ubiquinone, a component of the electron transport chain (Ewbank *et al*., 1997; Miyadera *et al*., 2001). *clk-1* mutant worms live longer than their WT counterparts (Lakowski & Hekimi, 1996) and their long lifespan is not further extended by the *eat-2* mutation (Lakowski & Hekimi, 1998), suggesting that *clk-1* is necessary for *eat-2* induced lifespan extension. Although the *clk-1* allele, *clk-1*(*e2519*), is unlikely to be a null mutant (Lakowski & Hekimi, 1996), we tested if *clk-1* was important for sDR-induced lifespan extension. We found that *clk-1*(*e2519*) mutant worms, similarly to *aak-2*(*ok524*) and *aak-2*(*rr48*) mutant worms, no longer responded to sDR (Fig. 5; Table S9). These results suggest that *clk-1* is necessary for sDR-induced longevity and are compatible with the observation that *clk-1* longevity like sDR-induced lifespan is dependent on *daf-16*. Although the interpretation of these results is difficult because of the lack of a null allele for *clk-1* (Gems *et al*., 2002), *clk-1* may mediate two independent methods of DR, *eat-2* and sDR. Thus, in addition to the genes that are specific to DR methods, there may also exist overlapping mechanisms underlying DR-induced longevity.

### The effects of sDR and *eat-2* on lifespan are additive

**Fig. 6:**
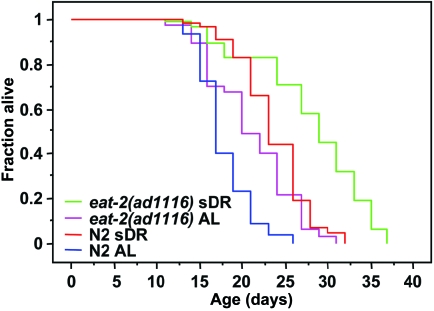
sDR and *eat-2* have an additive effect on lifespan. sDR extended WT (N2) worm lifespan (18.2%*P* < 0.0001) and *eat-2*(*ad1116*) mutant worm lifespan (18.0%*P* < 0.0001) to the same extent. sDR: 5 × 10^8^ bacteria mL^−1^ and AL (*ad libitum*): 5 × 10^11^ bacteria mL^−1^. Mean, standard errors, and statistical analysis for four independent experiments performed in triplicate are presented in Table S4.

The observation that sDR is mediated by AMPK, FoxO, and *clk-1* whereas *eat-2* is mediated by FoxA and *clk-1*, raised two possibilities: (i) *clk-1* is a common mechanism between both methods of DR but each method also triggers specific pathways in parallel; and (ii) each DR regimen is sensed by different pathways (e.g. by FoxO vs. FoxA), which both converge on *clk-1*. To distinguish between these two possibilities and to test whether sDR and *eat-2* had additive effects on longevity, we tested the combined effect of sDR and *eat-2* on lifespan. We found that sDR further extended the long lifespan of *eat-2* mutant worms (Fig. 6, Table S4). Thus, both DR regimens are additive and can extend lifespan by up to 57% when combined. Although the *eat-2* mutation is not a null mutation, which renders the interpretation of these experiments more difficult, these findings also suggest that *eat-2* and sDR evoke mostly independent, though overlapping, pathways to extend lifespan.

## Discussion

**Fig. 7:**
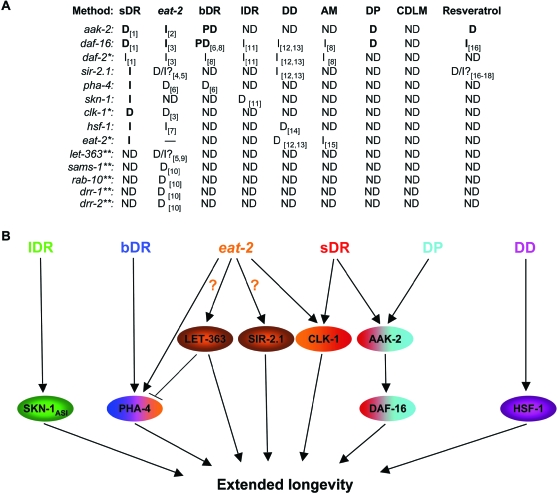
Different DR methods. (A) Table summarizing genes that have been tested for specific DR methods. Question marks indicate conflicting reports in the literature. sDR, ‘solid DR’ (Greer *et al*., 2007); *eat-2* (Lakowski & Hekimi, 1998); bDR, liquid DR (Klass, 1977; Panowski *et al*., 2007); lDR, Liquid DR (Bishop & Guarente, 2007); DD, dietary deprivation (Kaeberlein *et al*., 2006; Lee *et al*., 2006); AM, axenic medium (Houthoofd *et al*., 2002a; Houthoofd *et al*., 2002b); DP, dilution of peptone in plates (Hosono *et al*., 1989); CDLM, chemically defined liquid media (Szewczyk *et al*., 2006). D, dependent; PD, partially dependent; I, independent; ND, not determined. *Not a null mutant, making results more difficult to interpret. **Experiments were performed with RNAi, making results more difficult to interpret. *let-363*: *C. elegans* TOR gene mutant (Henderson *et al*., 2006; Hansen *et al*., 2007). [1]: (Greer *et al*., 2007), [2]: (Curtis *et al*., 2006), [3]: (Lakowski & Hekimi, 1998), [4]: (Wang & Tissenbaum, 2006), [5]: (Hansen *et al*., 2007), [6]: (Panowski *et al*., 2007), [7]: (Hsu *et al*., 2003), [8]: (Houthoofd *et al*., 2003) [9]: (Henderson *et al*., 2006), [10]: (Hansen *et al*., 2005), [11]: (Bishop & Guarente, 2007), [12]: (Kaeberlein *et al*., 2006), [13]: (Lee *et al*., 2006), [14]: (Steinkraus *et al*., 2008), [15]: (Houthoofd *et al*., 2002b), [16]: (Viswanathan *et al*., 2005), [17]: (Wood *et al*., 2004), [18]: (Bass *et al*., 2007).(B) Different methods of DR activate distinct signaling pathways. Displayed are molecules that have been shown to play a role in mediating the longevity extension effects of DR methods. Question marks indicate conflicting reports (Henderson *et al*., 2006; Wang & Tissenbaum, 2006; Hansen *et al*., 2007). LET-363: *C. elegans* TOR protein.

In this study, we performed a side-by-side comparison of the role of different genes in lifespan extension elicited by a variety of DR regimens. Our results uncover the importance of the low energy-sensing protein kinase, AMPK, in longevity due to some forms of DR and to resveratrol, a compound that extends lifespan and mimics some aspects of DR in many species. In addition, our findings provide further evidence that, contrary to the assumption that DR is independent of the insulin–FoxO pathway in invertebrates, the FoxO transcription factor *daf-16* actually plays a role in DR-induced longevity, depending on how DR is elicited (Fig. 7A). Importantly, our results show that DR is not a uniform condition that triggers a universal and linear genetic pathway. Rather, diverse DR regimens evoke mostly independent genetic pathways, depending on how DR is achieved (Fig. 7B). Identifying the specific genes that mediate DR-induced longevity in *C. elegans* is likely to have important implications for other species, including yeast, flies, and mammals, because of the conservation of the genes studied and because of the existence of different DR regimens in these other species as well. Understanding the genetic network by which different DR methods extend lifespan should also help harness the effects of this environmental intervention on lifespan and healthspan and will be particularly important to achieve the full benefits of DR.

### Parameters that may affect the mechanisms of DR-induced longevity

Different DR regimens may evoke distinct genetic pathways because some nutrients may be more limiting than others depending on the DR method. For example, sDR might reduce carbohydrates more prominently than amino-acids, which would render it dependent on AMPK and FoxO, while other methods (*eat-2*, bDR) may reduce amino-acids more readily, which would evoke TOR, a well-known amino-acid responsive pathway (Avruch *et al*., 2006), and the FoxA/*pha-4* transcription factor, which has recently been found to be downstream of TOR (Sheaffer *et al*., 2008). DR could also trigger different genetic mechanisms depending on the time at which DR is initiated or the tissues/cells by which it is sensed.

Alternatively, different DR regimens may evoke independent pathways because the way in which DR is initiated may trigger other non-DR signaling pathways that in turn have beneficial effects on lifespan. For example, diluting bacteria in sDR might also lead to the reduction in bacterial pathogenicity, which could in turn affect the insulin-FoxO pathway. However, if pathogenicity were the sole cause for premature death, concentrating bacteria above the *ad libitum* concentration would be detrimental, which we have found not to be the case. We have also shown that sDR is not simply due to a reduction in bacterial pathogenicity because sDR reduces worm fertility (data not shown) and because sDR performed with UV-killed bacteria also extends lifespan (Greer *et al*., 2007). The way sDR is achieved (by adding diluted amounts of *E. coli* every 2 days) may induce cycles of feeding and fasting that may cause this method to be AMPK and FoxO dependent whereas other DR methods (*eat-2*) maintain a more constant bacteria concentration throughout lifespan. sDR may also induce a mild oxidative stress response that could trigger the activation of the AMPK and FoxO pathways, as these pathways are known to transduce stress stimuli (Henderson & Johnson, 2001; Apfeld *et al*., 2004; Schulz *et al*., 2007; Lee *et al*., 2008). Although such stress may be part of DR itself (see the ‘hormesis theory’ of DR (Sinclair, 2005)), it is possible that the other DR methods do not require oxidative stress to extend lifespan.

Conversely, other methods of inducing DR could trigger non-DR signaling pathways. For example, the *eat-2* mutation, which affects the acetylcholine receptor, may have other effects in addition to reducing pharyngeal pumping. Liquid methods of DR may also evoke other parameters (e.g. altered oxygen consumption (Honda *et al*., 1993) or forced exercise (Retzlaff *et al*., 1966) since worms actively swim in liquid) that may interact with reduction in nutrients to result in lifespan extension in a manner that is dependent on specific genes. Identifying the parameters that are embedded in or interact with DR methods will be important to gain complete insight into the mechanisms by which DR extends lifespan.

### Mechanisms of DR in other species

An important question is whether the genetic mechanisms identified to regulate longevity in response to DR in *C. elegans* are conserved between species. The existence of different DR regimens that extend lifespan in yeast, flies, and mice raises the possibility that some of the mechanisms sensing the restriction of specific nutrients are conserved throughout evolution.

For example, in flies, a number of different methods of restricting diet extend lifespan (for a comprehensive review see (Piper & Partridge, 2007)). Two methods of DR in flies, dilution of yeast (Min *et al*., 2008) and dilution of both sugar and yeast (Giannakou *et al*., 2008) have been found to be independent of the *Drosophila* FoxO transcription factor, dFOXO, although dFOXO modifies the response to DR (Giannakou *et al*., 2008). While the role of AMPK in DR has not been tested yet, the histone deacetylases Rpd3 and Sir2, and the tumor suppressors Dmp53 and Tsc2, a member of the TOR pathway, have all been shown to be necessary for lifespan extension in different methods of DR in flies (Rogina *et al*., 2002; Kapahi *et al*., 2004; Rogina & Helfand, 2004; Bauer *et al*., 2005). The main method in which DR is initiated in flies, which reduces amino-acids, may primarily evoke the TOR and FoxA pathway rather than the AMPK or FoxO pathway (Kapahi *et al*., 2004).

In mice, reduction of the total amount of food which mice receive every day (Weindruch *et al*., 1986) or alternating days of feeding and fasting (EOD) both extend lifespan (Goodrick *et al*., 1990). The specific genetic components involved in DR in mice have not been as extensively analyzed yet, although the deletion of Sirt1, the mouse Sir2 ortholog, abrogates the beneficial effect of DR on behavioral activity (Chen *et al*., 2005) and mice expressing additionally copies of the Sirt1 gene have metabolic parameters similar to those induced by DR (Bordone *et al*., 2007). A reduction of the protein concentration (Goodrick, 1978) or of the amount of methionine in the diet also extend mouse lifespan (Orentreich *et al*., 1993), raising the possibility that the TOR pathway might be critical in longevity induced by these methods. While the importance of AMPK or FoxO in longevity induced by DR in mammals is still unknown, emerging evidence suggests that these pathways play some role in DR. First, EOD and a 40% restriction of food activate AMPK in the liver of rats (Pallottini *et al*., 2004). Short term DR (60% reduction of food for 5 days) also activated AMPK in the hippocampus of mice (Dagon *et al*., 2005). Second, the lifespan of mice that are deficient for the growth hormone receptor is not extended by a 30% reduction of food (Bonkowski *et al*., 2006). The deficiency in growth hormone receptor is thought to act via a reduction in IGF-1 and insulin levels, which would lead to FoxO activation. These observations raise the possibility that FoxO may mediate longevity in response to DR in mice.

### Role of AMPK in longevity induced by ‘DR mimetics’

Chemical compounds that mimic some of the beneficial effects of DR include 2-deoxyglucose (2DG) (Roth *et al*., 2001), metformin (Dhahbi *et al*., 2005), and resveratrol (Howitz *et al*., 2003). Metformin decreases the tumor incidence and extends the lifespan of tumor prone HER-2/neu transgenic mice (Anisimov *et al*., 2005). Resveratrol extends the lifespan of yeast (Howitz *et al*., 2003), worms (Wood *et al*., 2004; Viswanathan *et al*., 2005; Gruber *et al*., 2007), flies (Wood *et al*., 2004), fish (Valenzano *et al*., 2006) and mice on a high fat diet (Baur *et al*., 2006), although resveratrol does not extend lifespan under all circumstances (Bass *et al*., 2007; Pearson *et al*., 2008). Intriguingly, 2DG, resveratrol, and metformin have all been shown to activate AMPK (Baur *et al*., 2006; Zang *et al*., 2006; Dasgupta & Milbrandt, 2007; Hardie, 2007; Hwang *et al*., 2007). Resveratrol has been proposed to act through *sir-2.1* (Wood *et al*., 2004; Viswanathan *et al*., 2005), although this was not found in all studies (Bass *et al*., 2007), and AMPK/*aak-2* (this study) to affect lifespan, while 2DG was shown to act through AMPK/*aak-2*, but not *sir-2.1*, in *C. elegans* (Schulz *et al*., 2007). Thus, AMPK may play a pivotal role in mediating the lifespan extension induced by DR mimetics. However, the ‘DR mimetics’ have less of an impact on lifespan than DR itself. This observation suggests that multiple pathways need to be activated concomitantly to achieve optimal effects on lifespan and healthspan through chemical treatments.

### A gene network mediating longevity in response to DR

The pathways that regulate aging in response to DR are unlikely to be linear. Our findings indicate that resveratrol requires AMPK, but not FoxO, to extend lifespan, yet FoxO is necessary to mediate AMPK's effects on lifespan (Greer *et al*., 2007). Similarly, lifespan extension due to resveratrol is thought to be *sir-2.1* dependent (although not in all studies (Bass *et al*., 2007)) and *daf-16* independent (Wood *et al*., 2004; Viswanathan *et al*., 2005), yet the lifespan extension in response to *sir-2.1* overexpression is *daf-16* dependent (Tissenbaum & Guarente, 2001). These observations suggest that genetic pathways branching downstream of Sir-2 and AMPK probably exist and that negative and positive feedback mechanisms may also be involved. For example, AMPK substrates other than FoxO may contribute to AMPK lifespan extension, as proposed (Apfeld *et al*., 2004; Narbonne & Roy, 2006). The identification of additional AMPK substrates will be important for understanding the mechanisms of AMPK action on longevity. Our study may provide an explanation for the controversies regarding the implication of *daf-16* downstream of pro-longevity genes (Lakowski & Hekimi, 1996; Murakami & Johnson, 1996; Braeckman *et al*., 1999; Henderson *et al*., 2006; Wang & Tissenbaum, 2006; Hansen *et al*., 2007). Lifespan assay protocols might inadvertently induce sDR, which would render lifespan *daf-16* dependent. Thus, maintaining worms on *ad libitum* concentrations of food throughout lifespan experiments is likely crucial to prevent confounding variables, which could influence whether longevity is dependent on *daf-16*.

Finally, it is interesting to note that the genes involved in mediating different DR methods encode proteins from similar families (the Forkhead transcription factors FoxO and FoxA or the nutrient sensing kinases AMPK and TOR). In addition, the pathways mediating DR-induced longevity have already been shown to cross-talk extensively, at least in mammalian systems (e.g. AMPK and mTOR, Sir2 and AMPK) (Greer & Brunet, 2008). These observations underscore that DR-induced longevity is likely to be mediated by a network of genes rather than by linear pathways. Manipulating more than one ‘node’ in this network may allow additive or even synergistic lifespan and healthspan benefits.

## Materials and methods

### Worm strains and RNA interference

N2 and *daf-16*(*mu86*) strains were a kind gift from Dr Man-Wah Tan. The *aak-2*(*ok524*), *aak-2*(*rr48*), *sir-2.1*(*ok434*), *skn-1*(*zu135*), *hsf-1*(*sy441*), *clk-1*(*e2519*), *daf-16*(*mgDf50*), *daf-16*(*m26*), and *daf-16*(*mu86*), strains were provided by the *Caenorhabditis* Genetics Center. The *eat-2*(*ad1116*), *daf-16*(*mu86*); *eat-2*(*ad1116*), N2, and a three times backcrossed *aak-2(ok524)* strains were generously provided by Cynthia Kenyon. The *smg-1*(*cc546ts*) and *smg-1*(*cc546ts*); *pha-4*(*zu225*) strains were generously provided by Susan Mango. The *aak-2*(*ok524*) strain was crossed to *eat-2*(*ad1116*) to generate the double mutant strain *aak-2*(*ok524*); *eat-2*(*ad1116*). Unless otherwise noted, *aak-2*(*ok524*) and *daf-16*(*mu86*) strains were used. HT115 (DE3) bacteria transformed with vectors expressing dsRNA of the genes of interest were obtained from the Ahringer library (a gift from Dr M.-W. Tan) and were grown at 37 °C and seeded onto standard nematode growth medium (NGM) plates containing Ampicillin (100 µg mL^−1^) and IPTG (0.4 mm). Adult worms were placed on standard NGM plates and removed after 4–6 h to obtain synchronized populations of worms. L1 or L4 worms obtained from these synchronized populations were placed on NGM plates containing Ampicillin (100 µg mL^−1^) and IPTG (0.4 mm) seeded with the respective bacteria. Each vector was sequenced to verify the presence of the appropriate gene of interest.

### Lifespan assays

Worm lifespan assays were performed at 20 °C unless noted differently. Worm populations were synchronized by placing young adult worms on NGM plates seeded with the *E. coli* strain OP50–1 (unless otherwise noted) for 4–6 h and then removed. OP50–1 is a strain derived from OP50 that contains a streptomycine resistance gene (Kawli & Tan, 2008). The hatching day was counted as day 1 for all lifespan measurements. Worms were changed every other day to new plates to eliminate confounding progeny, and were marked as dead or alive. Worms were scored as dead if they did not respond to repeated prods with a platinum pick. Worms were censored if they crawled off the plate or died from vulval bursting. For each lifespan assay, 90 worms per condition were used in three plates (30 worms per plate). The data were plotted in StatView 5.0.1 using the Kaplan–Meier Survival curves and statistical significance was determined by Log-rank (Mantel-Cox) tests. Lifespan assays were repeated at least once unless otherwise mentioned. Representative Kaplan-Meier survival curves are shown in the figures. For lifespan assays performed on a gradient of bacterial concentrations mean and standard error values were taken from Kaplan–Meier Survival curves plotted in StatView 5.0.1 and plotted in Excel. Representative curves are presented unless noted otherwise. For *smg-1* and *smg-1*; *pha-4* lifespan assays, worms were grown at permissive temperature 24 °C until the first day of adulthood when they were switched to 15 °C which allows for the degradation of *pha-4*(*zu225*) (Gaudet & Mango, 2002).

### DR assays

OP50–1 bacteria were serially diluted from 5 × 10^12^ to 5 × 10^4^ bacteria mL^−1^. Bacteria were resuspended in S Medium to inhibit bacterial growth. Adult worms were placed on these various concentrations of bacteria starting at day 7 of life (day 4 of adulthood). Worms placed on a gradient of bacterial concentrations ranging from 5 × 10^4^ to 5 × 10^7^ bacteria mL^−1^ died 2 days after being placed on these extreme DR diets. For specific assays, sDR was considered 5 × 10^8^ bacteria mL^−1^ and *ad libitum* was 5 × 10^11^ bacteria mL^−1^ (Greer *et al*., 2007).

### Peptone dilution assays

Assays were performed as in (Hosono *et al*., 1989). Synchronized populations of worms were obtained by placing adult worms on plates with decreasing concentrations of peptone (from 2.5 to .0025 g L^−1^ with 150 µL of 5 × 10^12^ bacteria mL^−1^ seeded on each plate) and removing the worms after 4–6 h. Worms were switched every other day to fresh plates and were scored as alive as described above.

### Liquid DR assays

Assays were performed as in (Panowski *et al*., 2007). Briefly worms were grown until day 1 of adulthood on NGM plates seeded with 150 µL of 5 × 10^11^ bacteria mL^−1^. They were then transferred for 1 day to NGM plates with FUdR (100 mg L^−1^) seeded with 150 µL of 5 × 10^11^ bacteria mL^−1^. Approximately 20 worms were then placed in each well of a 12-well plate with 1 mL of S-basal supplemented with cholesterol (5 mg L^−1^), Ampicillin (50 µg L^−1^), Kanamycin (10 µg L^−1^), Tetracycline (1 µg L^−1^) and FUdR (100 mg L^−1^) containing OP50–1 bacteria at various concentrations (from 3.33 × 10^9^ to 5 × 10^11^ bacteria mL^−1^). Approximately 90 total worms were used for each condition (4 wells containing approximately 22 worms per well). Plates were gently shaken at 20 °C and worms were scored as alive as described before. Live worms were switched to fresh liquid medium containing the appropriate dilution of bacteria every third day.

### Statistical analysis

Statistical analysis of lifespan was performed on Kaplan–Meier survival curves in StatView 5.0.1. For statistical comparison of independent replicates, Fischer's combined probability tests were performed. To compare the interaction between genotype and food concentration, two-way anova tests were performed in Prism 4.0 c using the mean and standard error values obtained from the Kaplan–Meier survival curves. To compare the interaction between genotype and food concentration in an independent manner, regression analyses were also performed on raw lifespan data using a Cox proportional hazard analysis in r 2.7.1. The values from the Fisher's combined probability tests, two-way anova, and Cox proportional hazard analysis are included in the supplemental tables.

## Additional Note

Since this manuscript was accepted, another method of DR in C. elegans, termed IF for intermittent fasting, has been reported. This DR method is dependent on RHEB-1 and FoxO/*daf-16*, but independent of AMPK/*aak-2* (Honjoh S, Yamamoto T, Uno M, Nishida E (2009) Signalling through RHEB-1 mediates intermittent fasting-induced longevity in C. elegans. Nature 457, 726–730.)

## Supporting Information

Figure S1 Addition of FUdR does not alter the dependency of sDR-induced lifespan extension on AMPK/*aak-2* or FoxO/*daf-16*. sDR extended WT (N2) worm lifespan (31.0% *P* < 0.0001) but did not significantly extend *aak-2*(ok524) mutant worm lifespan (2.3% *P* = 0.3295) or *daf-16(mu86)* mutant worm lifespan (4.3% *P* = 0.0624) on plates with 100 mg L^-1^ FUdR. Mean, standard errors, and statistical analysis for one experiment performed in triplicate are presented in Table S1C.

Figure S2 AMPK and FoxO are not completely necessary for bDR lifespan extension. (A) In one experiment, bDR extended WT (N2) worm lifespan (101%, *P* < 0.0001), *aak-2*(ok524) mutant lifespan (47.3%, *P* < 0.0001), and *daf-16(mu86)* mutant lifespan (39.6%, *P* < 0.0001). Two-way ANOVA revealed that the lifespan extension of WT (N2) worms across a bacterial gradient was significantly different from that of *aak-2*(ok524) mutant worms (P < 0.0001) or *daf-16(mu86)* mutant worms (P < 0.0001). (B) In a second experiment, bDR extended WT (N2) worm lifespan (59.1%, *P* < 0.0001), *aak-2*(ok524) mutant lifespan (27.4%, P<0.0001), and *daf-16(mu86)* mutant lifespan (14.6%, *P* = 0.0120). Two-way ANOVA revealed that the lifespan extension of WT (N2) worms across a bacterial gradient was significantly different from that of *aak-2*(ok524) mutant worms (P < 0.0001) or *daf-16(mu86)* mutant worms (P < 0.0001). (C) In a third experiment, bDR extended WT (N2) worm lifespan (32.3%, P<0.0001), *aak-2*(ok524) mutant lifespan (20.8%, P<0.0001), and *daf-16(mu86)* mutant lifespan (26.7%,Two-way ANOVA revealed that the lifespan extension of WT (N2) worms across a bacterial gradient was not significantly different from that of *aak-2*(ok524) mutant worms (*P* = 0.1528) and *daf-16(mu86)* mutant worms (*P* = 0.6643). Mean, standard errors, and statistical analysis for these experiments performed in quadruplicates are presented in Table S3.

Figure S3 FoxA/pha-4 is not necessary for sDR to extend lifespan. (A) sDR extended the lifespan of WT (N2) worms treated with empty vector RNAi (E.V.) (34.7% *P* < 0.0001) or pha-4 RNAi (36.7% *P* < 0.0001) initiated at L1, but not the lifespan of worms treated with *daf-16* RNAi (7.0 %*P* = 0.1562). The efficacy of pha-4 RNAi was confirmed by its ability to decrease worm fertility, consistent with its known role in pharyngeal organogenesis (Mango et al., 1994), and its effects on lifespan (Panowski et al., 2007). Mean, standard errors, and statistical analysis for two independent experiments performed in triplicate are presented in Table S7B. (B) sDR extended the lifespan of WT (N2) worms treated with empty vector RNAi (E.V.) (30.8% *P* < 0.0001) or pha-4 RNAi (26.4% *P* < 0.0001) initiated at L4 but not the lifespan of worms treated with *daf-16* RNAi (6.1 *P* = 0.1801). Mean, standard errors, and statistical analysis for two independent experiments performed in triplicate are presented in Table S7B.

**Table S1** AMPK/*aak-2* and FoxO/daf-16 are necessary for lifespan extension by sDR across a gradient of bacteria. (A) A serial dilution of bacteria extends WT (N2) worm lifespan but does not extend *aak-2*(ok524) mutant worm lifespan. Experiment #1 is displayed in Figure 1A. Combined p values were calculated using Fisher's combined probability test. (B) A serial dilution of bacteria extends WT (N2) worm lifespan but does not extend *daf-16 (mu86)* mutant worm lifespan. This experiment is displayed in Figure 1B. (C) Addition of FUdR does not alter the dependency of sDR-induced lifespan extension on AMPK/*aak-2* or FoxO/daf- 16. This experiment is displayed in Figure S1. The mean lifespan values were calculated by a log-rank (Mantel-Cox) statistical test from triplicate samples of 30 worms each. n, number of observed dead worms/number of total worms.

**Table S2** Dilution of peptone (DP) extends lifespan in an AMPK/*aak-2* and FoxO/*daf-16* dependent manner. Experiment #2 is displayed in Figure 2A. The mean lifespan values were calculated by a log-rank (Mantel-Cox) statistical test from triplicate samples of 30 worms each. n, number of observed dead worms/number of total worms. Combined *P*-values were calculated using Fisher#x0027;s combined probability test.

**Table S3** bDR increase in worm lifespan is partially dependent on AMPK/*aak-2* and FoxO/*daf-16*. The average of these three experiments is displayed in Figure 2B. Each experiment is displayed in Figure S2. The mean lifespan values were calculated by a log-rank (Mantel-Cox) statistical test from quadruplicate samples of approximately 22 worms each. n, number of observed dead worms/number of total worms. Combined *P*-values were calculated using Fisher's combined probability test.

**Table S4** (A) *eat-2(ad1116)* induced lifespan extension is independent of AMPK/*aak-2*. Experiment #1 is displayed in Figure 2C. Experiment #2 is displayed in Figure 6. (B) *eat-2(ad1116)* induced lifespan extension is independent of FoxO/*daf-16*. Experiment #2 is displayed in Figure 2D. The mean lifespan values were calculated by a log-rank (Mantel-Cox) statistical test from triplicate samples of 30 worms each. n, number of observed dead worms/number of total worms. Combined Pvalues were calculated using Fisher'427s combined probability test.

**Table S5** Resveratrol increases worm lifespan in an AMPK/*aak-2* dependent but FoxO/*daf-16* independent manner.Experiment #2 is displayed in Figure 3A. Experiment #3 is displayed in Figure 3B. The mean lifespan values were calculated by a log-rank (Mantel-Cox) statistical test from triplicate samples of 30 worms each. n, number of observed dead worms/number of total worms. Combined *P*-values were calculated using Fisher's combined probability test.

**Table S6** sDR increases worm lifespan in an AMPK/*aak-2* dependent and SIR2/*sir-2.1* independent manner. Experiment #3 is displayed in Figure 4A. The mean lifespan values were calculated by a log-rank (Mantel-Cox) statistical test from triplicate samples of 30 worms each. *n*, number of observed dead worms/ number of total worms. Combined *P*-values were calculated using Fisher's combined probability test.

**Table S7** sDR increases worm lifespan in a FoxA/*pha-4* independent manner. A) *smg-1* and *smg-1*; *pha-4* worms were grown at permissive temperature (24 °C) until the first day of adulthood when they were switched to 15 °C. Experiment #1 is displayed in Figure 4B. B) sDR extends the lifespan of WT(N2) worms treated with empty vector RNAi (E.V.) or *pha-4* RNAi bute not *daf-16* RNAi initiated at larval stage L1 or larval stage L4. L1 Experiment #1 is displayed in Figure S3A. L4 Experiment #2 is displayed in Figure S3B. The mean lifespan values were calculated by a log-rank (Mantel-Cox) statistical test from triplicate samples of 30 worms each. n, number of observed dead worms/number of total worms. Combined *P*-values were calculated using Fisher's combined probability test.

**Table S8** sDR increases worm lifespan in an AMPK/*aak-2* dependent, skn-1 independent manner. Experiment #2 is displayed in Figure 4C. The mean lifespan values were calculated by a log-rank (Mantel-Cox) statistical test from triplicate samples of 30 worms each. n, number of observed dead worms/number of total worms. Combined *P*-values were calculated using Fisher's combined probability test.

**Table S9** sDR increases worm lifespan in an AMPK/*aak-2* and clk-1 dependent, hsf-1 independent manner. Experiment #2 is displayed in Figure 4C. Experiment #1 is displayed in Figure 5. The mean lifespan values were calculated by a log-rank (Mantel- Cox) statistical test from triplicate samples of 30 worms each. n: number of observed dead worms/number of total worms. Combined *P*-values were calculated using Fisher's combined probability test. Note that experiment #2 of Table S9 was performed at the same time as experiment #2 of Table S8.
