# Supplementary material for: Different dietary restriction regimens extend lifespan by both independent and overlapping genetic pathways in C. elegans
Source: Aging Cell. 2009 Apr;8(2):113–27. doi: 10.1111/j.1474-9726.2009.00459.x (PMC2680339; doi:10.1111/j.1474-9726.2009.00459.x)
Supplement: Supplementary file 4 [file ace0008-0113-SD4.pdf]

A

sDR-induced longevity is AMPK/*aak-2* dependent

Strain (bacteria conc. /ml)	1: Mean +/- SD	1: n	2: Mean +/- SD	2: n
N2 (5x10^{12})	17.260 +/- 0.479	82/90	22.257 +/- 0.652	66/90
N2 (5x10^{11})	18.462 +/- 0.630	74/90	21.369 +/- 0.634	57/70
N2 (5x10^{10})	19.526 +/- 0.807	61/90	22.910 +/- 0.634	65/90
N2 (5x10^{9})	21.940 +/- 0.628	68/90	25.597 +/- 0.722	48/90
N2 (5x10^{8})	24.065 +/- 0.773	47/90	26.641 +/- 0.728	48/90
N2 (5x10^{7})	18.361 +/- 0.748	42/90	19.461 +/- 1.673	12/75
aak-2(ok524) (5x10^{12})	15.363 +/- 0.358	84/90	16.616 +/- 0.615	66/90
aak-2(ok524) (5x10^{11})	14.944 +/- 0.376	69/90	16.441 +/- 0.459	63/67
aak-2(ok524) (5x10^{10})	14.767 +/- 0.349	68/90	16.946 +/- 0.428	62/90
aak-2(ok524) (5x10^{9})	14.843 +/- 0.408	58/90	16.537 +/- 0.415	46/90
aak-2(ok524) (5x10^{8})	14.691 +/- 0.391	57/90	17.151 +/- 0.440	55/90
aak-2(ok524) (5x10^{7})	14.827 +/- 0.413	55/90	16.610 +/- 0.756	22/70

Statistical comparison	1: p values	2: p values	Combined p values
N2 (5x10^{12})/N2 (5x10^{11})	0.0502	0.2198	0.0608
N2 (5x10^{11})/N2 (5x10^{10})	0.1439	0.0564	0.0472
N2 (5x10^{11})/N2 (5x10^{9})	0.0003	<0.0001	<0.0001
N2 (5x10^{11})/N2 (5x10^{8})	<0.0001	<0.0001	<0.0001
N2 (5x10^{11})/N2 (5x10^{7})	0.8426	0.2145	0.4900
aak-2 (5x10^{12})/*aak-2* (5x10^{11})	0.4320	0.9547	0.7778
aak-2 (5x10^{11})/*aak-2* (5x10^{10})	0.6034	0.5514	0.6988
aak-2 (5x10^{11})/*aak-2* (5x10^{9})	0.8143	0.9244	0.9665
aak-2 (5x10^{11})/*aak-2* (5x10^{8})	0.5955	0.3950	0.5757
aak-2 (5x10^{11})/*aak-2* (5x10^{7})	0.8233	0.9888	0.9815
N2 (5x10^{11})/*aak-2* (5x10^{11})	<0.0001	<0.0001	<0.0001
Interaction between genotype and food concentration			
Two-way ANOVA N2/*aak-2*	<0.0001	<0.0001	<0.0001
Cox proportional hazard N2/*aak-2*	<0.0001	<0.0001	<0.0001

B

sDR-induced longevity is FoxO/*daf-16* dependent

Strain (bacteria conc. /ml)	Mean +/- SD	n
N2 (5x10^{12})	21.836 +/- 0.703	77/90
N2 (5x10^{11})	21.982 +/- 0.656	72/90
N2 (5x10^{10})	24.042 +/- 0.620	85/90
N2 (5x10^{9})	27.084 +/- 0.891	57/90
N2 (5x10^{8})	27.600 +/- 0.754	75/90
N2 (5x10^{7})	17.659 +/- 1.318	33/90
daf-16(mu86) (5x10^{12})	15.056 +/- 0.321	72/90
daf-16(mu86) (5x10^{11})	15.111 +/- 0.277	66/90
daf-16(mu86) (5x10^{10})	14.834 +/- 0.358	56/90
daf-16(mu86) (5x10^{9})	15.048 +/- 0.327	60/90
daf-16(mu86) (5x10^{8})	15.068 +/- 0.395	44/90
daf-16(mu86) (5x10^{7})	13.495 +/- 0.374	44/90

Statistical comparison	p values
N2 (5x10^{12})/N2 (5x10^{11})	0.8143
N2 (5x10^{11})/N2 (5x10^{10})	0.0202
N2 (5x10^{11})/N2 (5x10^{9})	<0.0001
N2 (5x10^{11})/N2 (5x10^{8})	<0.0001
N2 (5x10^{11})/N2 (5x10^{7})	0.0040
daf-16 (5x10^{12})/*daf-16* (5x10^{11})	0.9476
daf-16 (5x10^{11}) /*daf-16* (5x10^{10})	0.6192
daf-16 (5x10^{11})/*daf-16* (5x10^{9})	0.9444
daf-16 (5x10^{11}) /*daf-16* (5x10^{8})	0.7805
daf-16 (5x10^{11})/*daf-16* (5x10^{7})	0.0008
N2 (5x10^{11})/*daf-16* (5x10^{11})	<0.0001
Interaction between genotype and food concentration	
Two-way ANOVA N2/*daf-16*	<0.0001
Cox proportional hazard N2/*daf-16*	<0.0001

C

sDR extends lifespan in presence of FUdR

Strain (treatment)	Mean +/- SD	n
N2 (AL)	20.756 +/- .298	90/90
N2 (sDR)	27.183 +/- .317	79/90
aak-2(ok524) (AL)	15.047 +/- .323	89/90
aak-2(ok524) (sDR)	15.400 +/- .365	90/90
daf-16(mu86) (AL)	15.077 +/- .240	89/90
daf-16(mu86) (sDR)	15.722 +/- .269	89/90

Statistical comparison	p values
N2 (AL)/N2 (sDR)	<0.0001
aak-2 (AL)/*aak-2* (sDR)	0.3295
daf-16 (AL)/*daf-16* (sDR)	0.0624
N2 (AL)/*aak-2* (AL)	<0.0001
N2 (AL)/*daf-16* (AL)	<0.0001

Table S1: AMPK/*aak-2* and FoxO/*daf-16* are necessary for lifespan extension by sDR across a gradient of bacteria. A) A serial dilution of bacteria extends WT (N2) worm lifespan but does not extend *aak-2(ok524)* mutant worm lifespan. Experiment #1 is displayed in Figure 1A. Combined p values were calculated using Fisher's combined probability test. B) A serial dilution of bacteria extends WT (N2) worm lifespan but does not extend *daf-16(mu86)* mutant worm lifespan. This experiment is displayed in Figure 1B. C) Addition of FUdR does not alter the dependency of sDR-induced lifespan extension on AMPK/*aak-2* or FoxO/*daf-16*.This experiment is displayed in Figure S1. The mean lifespan values were calculated by a logrank (Mantel-Cox) statistical test from triplicate samples of 30 worms each. n: number of observed dead worms/number of total worms.

Dilution of peptone-induced longevity is *aak-2* and *daf-16* dependent	1		2		
**Strain (peptone conc. g/l)**	**Mean +/- SD**	**n**	**Mean +/- SD**	**n**	
N2 (2.5)	18.204 +/- 0.502	86/90	21.067 +/- 0.513	85/90	
N2 (0.25)	19.860 +/- 0.509	82/90	23.421 +/- 0.532	87/90	
N2 (0.025)	22.365 +/- 0.534	69/90	24.188 +/- 0.561	86/90	
N2 (0.0025)	22.888 +/- 0.576	65/90	26.409 +/- 0.486	88/90	
aak-2(ok524) (2.5)	14.837 +/- 0.388	76/90	15.128 +/- 0.405	89/90	
aak-2(ok524) (0.25)	14.617 +/- 0.374	89/90	14.710 +/- 0.352	89/90	
aak-2(ok524) (0.025)	15.084 +/- 0.354	89/90	15.356 +/- 0.421	90/90	
aak-2(ok524) (0.0025)	15.103 +/- 0.377	87/90	14.978 +/- 0.377	90/90	
daf-16(mu86) (2.5)	15.743 +/- 0.372	86/90	15.750 +/- 0.418	89/90	
daf-16(mu86) (0.25)	15.756 +/- 0.352	90/90	16.356 +/- 0.438	90/90	
daf-16(mu86) (0.025)	16.565 +/- 0.355	89/90	16.651 +/- 0.457	89/90	
daf-16(mu86) (0.0025)	16.427 +/- 0.361	89/90	16.644 +/- 0.434	90/90	
**Statistical comparison**	**p values**		**p values**	**Combined p values**	
N2 (2.5)/N2 (0.0025)	<0.0001		<0.0001	<0.0001	
N2 (2.5)/N2 (0.025)	<0.0001		<0.0001	<0.0001	
N2 (2.5)/N2 (0.25)	<0.0001		0.0015	<0.0001	
aak-2 (2.5)/*aak-2* (0.0025)	0.6351		0.6371	0.7707	
aak-2 (2.5)/*aak-2* (0.025)	0.6778		0.7443	0.8497	
aak-2 (2.5)/*aak-2* (0.25)	0.7021		0.3337	0.5744	
daf-16 (2.5)/*daf-16* (0.0025)	0.1899		0.1402	0.1232	
daf-16 (2.5)/*daf-16* (0.025)	0.1637		0.1191	0.0963	
daf-16 (2.5)/*daf-16* (0.25)	0.9842		0.3246	0.6840	
N2 (2.5)/*daf-16* (2.5)	<0.0001		<0.0001	<0.0001	
N2 (2.5)/*aak-2* (2.5)	<0.0001		<0.0001	<0.0001	
N2 (0.0025)/*aak-2* (0.0025)	<0.0001		<0.0001	<0.0001	
N2 (0.0025)/*daf-16* (0.0025)	<0.0001		<0.0001	<0.0001	
Interaction between genotype and food concentration					
Two-way ANOVA N2/*aak-2*	<0.0001		<0.0001	<0.0001	
Two-way ANOVA N2/*daf-16*	<0.0001		<0.0001	<0.0001	
Cox proportional hazard N2/*aak-2*	<0.0001		<0.0001	<0.0001	
Cox proportional hazard N2/*daf-16*	<0.0001		0.0001	<0.0001	

Table S2: Dilution of peptone (DP) extends lifespan in an AMPK/*aak-2* and FoxO/*daf-16* dependent manner. Experiment #2 is displayed in Figure 2A. The mean lifespan values were calculated by a logrank (Mantel-Cox) statistical test from triplicate samples of 30 worms each. n: number of observed dead worms/number of total worms. Combined p values were calculated using Fisher's combined probability test.

bDR extends worm lifespan	1		2		3		
**Strain (bacteria conc. /ml)**	**Mean +/- SD**	**n**	**Mean +/- SD**	**n**	**Mean +/- SD**	**n**	
N2 ($5x10^{11}$)	21.567 +/- 0.951	93/99	28.424 +/- 0.886	76/87	33.514 +/- 1.173	87/89	
N2 ($1x10^{11}$)	29.952 +/- 0.981	70/82	43.089 +/- 1.454	79/79	40.603 +/- 1.311	93/94	
N2 ($5x10^{10}$)	36.518 +/- 0.804	85/89	45.221 +/- 1.620	82/83	44.328 +/- 1.669	92/98	
N2 ($1.67x10^{10}$)	37.911 +/- 1.106	83/89	41.851 +/- 1.353	87/87	39.115 +/- 1.406	81/82	
N2 ($3.33x10^{9}$)	43.466 +/- 1.334	79/91	40.311 +/- 1.356	74/74	38.459 +/- 1.316	88/89	
aak-2(ok524) ($5x10^{11}$)	19.683 +/- 0.635	98/110	22.449 +/- 0.673	84/96	30.486 +/- 0.841	60/81	
aak-2(ok524) ($1x10^{11}$)	25.400 +/- 0.600	88/90	25.472 +/- 1.052	78/79	31.920 +/- 0.890	87/87	
aak-2(ok524) ($5x10^{10}$)	27.781 +/- 0.719	81/89	28.596 +/- 0.640	74/78	36.832 +/- 0.896	80/82	
aak-2(ok524) ($1.67x10^{10}$)	25.513 +/- 0.760	82/91	23.753 +/- 0.911	65/71	33.903 +/- 0.844	84/86	
aak-2(ok524) ($3.33x10^{9}$)	28.989 +/- 0.593	87/88	23.850 +/- 0.895	68/75	32.776 +/- 0.927	85/85	
daf-16(mu86) ($5x10^{11}$)	20.497 +/- 0.424	103/106	21.817 +/- 1.062	46/49	27.062 +/- 0.838	78/81	
daf-16(mu86) ($1x10^{11}$)	22.474 +/- 0.564	85/88	22.109 +/- 1.214	50/51	32.473 +/- 0.951	93/94	
daf-16(mu86) ($5x10^{10}$)	24.504 +/- 0.591	85/88	24.997 +/- 0.993	65/72	34.286 +/- 1.045	92/98	
daf-16(mu86) ($1.67x10^{10}$)	24.785 +/- 0.497	85/89	21.955 +/- 0.775	67/67	30.791 +/- 0.878	81/82	
daf-16(mu86) ($3.33x10^{9}$)	28.619 +/- 0.424	78/88	21.822 +/- 1.021	53/62	29.452 +/- 0.964	90/94	
**Statistical comparison**	**p values**		**p values**		**p values**		**Combined p values**
N2 (AL)/N2 (DR)	<0.0001		<0.0001		<0.0001		<0.0001
aak-2 (AL)/*aak-2* (DR)	<0.0001		<0.0001		<0.0001		<0.0001
daf-16 (AL)/*daf-16* (DR)	<0.0001		0.0120		<0.0001		<0.0001
Interaction between genotype and food concentration							
Two-way ANOVA N2/*aak-2*	<0.0001		<0.0001		0.1528		<0.0001
Two-way ANOVA N2/*daf-16*	<0.0001		<0.0001		0.6643		<0.0001
Cox proportional hazard N2/*aak-2*	<0.0001		0.0299		0.2490		<0.0001
Cox proportional hazard N2/*daf-16*	<0.0001		0.0024		0.9500		<0.0001

Table S3: bDR increase in worm lifespan is partially dependent on AMPK/*aak-2* and FoxO/*daf-16*. The average of these three experiments is displayed in Figure 2B. Each experiment is displayed in Figure S2. The mean lifespan values were calculated by a logrank (Mantel-Cox) statistical test from quadruplicate samples of ~22 worms each. n: number of observed dead worms/number of total worms. Combined p values were calculated using Fisher's combined probability test.

A

eat-2 longevity is independent of AMPK/*aak-2*	1		2	
**Strain (treatment)**	**Mean +/- SD**	**n**	**Mean +/- SD**	**n**
N2 (AL)	21.699 +/- .553	86/90	17.887 +/- .341	78/90
N2 (sDR)	25.639 +/- .687	71/90	23.676 +/- .590	44/90
aak-2(ok524) (AL)	17.581 +/- .461	88/90	14.788 +/- .286	68/90
aak-2(ok524) (sDR)	17.819 +/- .449	85/90	15.029 +/- .304	69/90
eat-2(ad1116) (AL)	25.993 +/- .705	84/90	21.097 +/- .688	44/90
eat-2(ad1116) (sDR)	30.682 +/- .813	65/90	28.053 +/- 1.212	22/90
eat-2(ad1116); aak-2(ok524) (AL)	20.983 +/- .569	76/90	18.359 +/- .336	73/90
eat-2(ad1116); aak-2(ok524) (sDR)	20.827 +/- .530	76/90	18.566 +/- .427	53/90

**Statistical comparison**	**p values**	**p values**	**Combined p values**
N2 (AL)/N2 (sDR)	<0.0001	<0.0001	<0.0001
eat-2 (AL)/*eat-2* (sDR)	<0.0001	<0.0001	<0.0001
aak-2 (AL)/*aak-2* (sDR)	0.8252	0.5985	0.8423
eat-2; aak-2 (AL)/*eat-2; aak-2* (sDR)	0.7449	0.7531	0.8853
N2 (AL)/*eat-2* (AL)	<0.0001	0.0002	<0.0001
N2 (AL)/*aak-2* (AL)	<0.0001	<0.0001	<0.0001
N2 (AL)/*eat-2; aak-2* (AL)	0.4351	0.3245	0.4176
N2 (sDR)/*eat-2* (sDR)	<0.0001	0.0034	<0.0001
N2 (sDR)/*aak-2* (sDR)	<0.0001	<0.0001	<0.0001
N2 (sDR)/*eat-2; aak-2* (sDR)	<0.0001	<0.0001	<0.0001
eat-2 (AL)/*eat-2; aak-2* (AL)	<0.0001	0.0064	<0.0001
eat-2 (sDR)/*eat-2; aak-2* (sDR)	<0.0001	<0.0001	<0.0001
aak-2 (AL)/*eat-2; aak-2* (AL)	<0.0001	<0.0001	<0.0001
aak-2 (sDR)/*eat-2; aak-2* (sDR)	<0.0001	<0.0001	<0.0001

B

eat-2 longevity is independent of FoxO/*daf-16*	1		2	
**Strain (treatment)**	**Mean +/- SD**	**n**	**Mean +/- SD**	**n**
N2 (AL)	21.39 +/- 0.75	88/90	20.36 +/- 0.45	87/90
N2 (sDR)	26.11 +/- 0.53	83/90	26.10 +/- 0.64	54/90
eat-2(ad1116) (AL)	24.15 +/- 0.58	86/90	22.21 +/- 0.59	77/90
eat-2(ad1116) (sDR)	27.16 +/- 0.66	64/90	26.39 +/- 0.72	68/90
daf-16(mu86) (AL)	16.53 +/- 0.33	90/90	14.71 +/- 0.29	87/90
daf-16(mu86) (sDR)	17.16 +/- 0.38	89/90	15.34 +/- 0.35	82/90
eat-2(ad1116); daf-16(mu86) (AL)	21.87 +/- 0.55	86/90	17.67 +/- 0.46	83/90
eat-2(ad1116); daf-16(mu86) (sDR)	21.81 +/- 0.52	86/90	17.70 +/- 0.50	82/90

**Statistical comparison**	**p values**	**p values**	**Combined p values**
N2 (AL)/N2 (sDR)	<0.0001	<0.0001	<0.0001
eat-2 (AL)/*eat-2* (sDR)	0.0010	<0.0001	<0.0001
daf-16 (AL)/*daf-16* (sDR)	0.1948	0.0798	0.0803
eat-2; daf-16 (AL)/*eat-2; daf-16* (sDR)	0.9574	0.7979	0.9696
N2 (AL)/*eat-2* (AL)	<0.0001	0.0007	<0.0001
N2 (AL)/*daf-16* (AL)	<0.0001	<0.0001	<0.0001
N2 (AL)/*eat-2; daf-16* (AL)	0.1480	<0.0001	0.0002
N2 (sDR)/*eat-2* (sDR)	0.1179	0.3430	0.1702
N2 (sDR)/*daf-16* (sDR)	<0.0001	<0.0001	<0.0001
N2 (sDR)/*eat-2; daf-16* (sDR)	<0.0001	<0.0001	<0.0001
eat-2 (AL)/*eat-2; daf-16* (AL)	0.0059	<0.0001	<0.0001
eat-2 (sDR)/*eat-2; daf-16* (sDR)	<0.0001	<0.0001	<0.0001
daf-16 (AL)/*eat-2; daf-16* (AL)	<0.0001	<0.0001	<0.0001
daf-16 (sDR)/*eat-2; daf-16* (sDR)	<0.0001	<0.0001	<0.0001

Table S4: A) *eat-2(ad1116*) induced lifespan extension is independent of AMPK/*aak-2*. Experiment #1 is displayed in Figure 2C. Experiment #2 is displayed in Figure 6. B) *eat-2(ad1116*) induced lifespan extension is independent of FoxO/*daf-16*. Experiment #2 is displayed in Figure 2D. The mean lifespan values were calculated by a logrank (Mantel-Cox) statistical test from triplicate samples of 30 worms each. n: number of observed dead worms/number of total worms. Combined p values were calculated using Fisher's combined probability test.

Table S5

**Resveratrol extends worm lifespan**	1		2		3	
**Strain (Resveratrol conc.)**	**Mean +/- SD**	**n**	**Mean +/- SD**	**n**	**Mean +/- SD**	**n**
N2 (0μM)	20.161 +/- .528	89/90	18.393 +/- .448	81/90	21.615 +/- .535	89/90
N2 (20μM)	21.845 +/- .503	89/90	21.461 +/- .484	76/90		
N2 (100μM)	23.028 +/- .537	84/90	21.698 +/- .486	71/90	24.774 +/- .575	88/90
N2 (500μM)	22.219 +/- .540	76/90	21.627 +/- .495	75/90		
aak-2(ok524) (0μM)	15.042 +/- .348	75/90	15.043 +/- .262	84/90	17.174 +/- .417	84/90
aak-2(ok524) (20μM)	14.464 +/- .367	73/90	15.897 +/- .297	87/90		
aak-2(ok524) (100μM)	15.370 +/- .383	64/90	15.375 +/- .316	82/90	15.686 +/- .381	75/90
aak-2(ok524) (500μM)	14.662 +/- .429	41/90	15.561 +/- .288	83/90		
daf-16(mu86) (0μM)					15.083 +/- .319	84/90
daf-16(mu86) (100μM)					17.151 +/- .379	85/90
**Statistical comparison**	**p values**		**p values**		**p values**	**Combined p values**
N2 (0μM)/N2 (20μM)	0.0484		<0.0001			<0.0001
N2 (0μM)/N2 (100μM)	0.0005		<0.0001		<0.0001	<0.0001
N2 (0μM)/N2 (500μM)	0.0159		<0.0001			<0.0001
aak-2 (0μM)/*aak-2* (20μM)	0.3095		0.0225			0.0416
aak-2 (0μM)/*aak-2* (100μM)	0.5485		0.2290		0.0096	0.0366
aak-2 (0μM)/*aak-2* (500μM)	0.5031		0.1126			0.2193
daf-16 (0μM)/*daf-16* (100μM)					<0.0001	
N2 (0μM)/*aak-2* (0μM)	<0.0001		<0.0001		<0.0001	<0.0001
N2 (0μM)/*daf-16* (0μM)					<0.0001	

Table S5: Resveratrol increases worm lifespan in an AMPK/*aak-2* dependent but FoxO/*daf-16* independent manner.Experiment #2 is displayed in Figure 3A. Experiment #3 is displayed in Figure 3B. The mean lifespan values were calculated by a logrank (Mantel-Cox) statistical test from triplicate samples of 30 worms each. n: number of observed dead worms/number of total worms. Combined p values were calculated using Fisher's combined probability test.

sDR-induced longevity is *sir-2.1* independent	1		2		3	
**Strain (bacteria conc. /ml)**	**Mean +/- SD**	**n**	**Mean +/- SD**	**n**	**Mean +/- SD**	**n**
N2 ($5x10^{12}$)					17.364 +/- 0.425	83/90
N2 ($5x10^{11}$)	19.041 +/- 0.566	84/90	19.041 +/- 0.546	85/90	17.139 +/- 0.445	72/90
N2 ($5x10^{10}$)					18.129 +/- 0.539	66/90
N2 ($5x10^{9}$)					20.301 +/- 0.589	62/90
N2 ($5x10^{8}$)	24.860 +/- 0.713	79/90	21.598 +/- 0.598	79/90	21.609 +/- 0.667	49/90
N2 ($5x10^{7}$)					16.329 +/- 0.557	66/90
aak-2(ok524) ($5x10^{12}$)					14.881 +/- 0.386	70/90
aak-2(ok524) ($5x10^{11}$)					14.971 +/- 0.389	68/90
aak-2(ok524) ($5x10^{10}$)					15.329 +/- 0.439	53/90
aak-2(ok524) ($5x10^{9}$)					15.572 +/- 0.436	49/90
aak-2(ok524) ($5x10^{8}$)					15.162 +/- 0.414	69/90
aak-2(ok524) ($5x10^{7}$)					14.077 +/- 0.432	55/90
sir-2.1(ok434) ($5x10^{12}$)					18.148 +/- 0.471	86/90
sir-2.1(ok434) ($5x10^{11}$)	19.423 +/- 0.382	76/90	17.812 +/- 0.344	76/90	18.138 +/- 0.473	79/90
sir-2.1(ok434) ($5x10^{10}$)					18.301 +/- 0.427	78/90
sir-2.1(ok434) ($5x10^{9}$)					19.179 +/- 0.527	66/90
sir-2.1(ok434) ($5x10^{8}$)	21.075 +/- 0.513	61/90	18.752 +/- 0.376	62/90	21.147 +/- 0.552	61/90
sir-2.1(ok434) ($5x10^{7}$)					18.017 +/- 0.612	53/90

**Statistical comparison**	**p values**	**p values**	**p values**	**Combined p values**
N2 ($5x10^{11}$)/N2 ($5x10^{8}$)	<0.0001	0.0019	<0.0001	<0.0001
aak-2 ($5x10^{11}$)/*aak-2* ($5x10^{8}$)			0.6330	
sir2.1 ($5x10^{11}$)/*sir-2.1* ($5x10^{8}$)	0.0214	0.0821	<0.0001	<0.0001
N2 ($5x10^{11}$)/*aak-2* ($5x10^{11}$)			0.0002	
N2 ($5x10^{11}$)/*sir-2.1* ($5x10^{11}$)	0.4583	0.0097	0.1111	0.0185
Interaction between genotype and food concentration				
Two-way ANOVA N2/*aak-2*			<0.0001	
Two-way ANOVA N2/*sir-2.1*	0.0002	0.0925	0.1240	0.0002
Cox proportional hazard N2/*aak-2*			0.0080	
Cox proportional hazard N2/*sir-2.1*			0.5322	

Table S6: sDR increases worm lifespan in an AMPK/*aak-2* dependent and SIR2/*sir-2.1* independent manner. Experiment #3 is displayed in Figure 4A. The mean lifespan values were calculated by a logrank (Mantel-Cox) statistical test from triplicate samples of 30 worms each. n: number of observed dead worms/number of total worms. Combined p values were calculated using Fisher's combined probability test.

A

**sDR-induced longevity is *pha-4* independent**	1		2	
**Strain (bacteria conc. /ml)**	**Mean +/- SD**	**n**	**Mean +/- SD**	**n**
smg-1(cc546ts) (5x10^{12})	25.675 +/- 0.930	60/90	19.789 +/- 0.530	90/90
smg-1(cc546ts) (5x10^{11})	27.348 +/- 0.854	80/90	21.240 +/- 0.556	85/90
smg-1(cc546ts) (5x10^{10})	31.601 +/- 1.040	72/90	25.141 +/- 1.004	61/90
smg-1(cc546ts) (5x10^{9})	33.103 +/- 1.395	51/90	28.454 +/- 1.046	59/90
smg-1(cc546ts) (5x10^{8})	32.241 +/- 1.114	54/90	22.518 +/- 0.993	68/90
smg-1(cc546ts) (5x10^{7})	23.566 +/- 1.139	60/90	20.306 +/- 0.819	72/90
smg-1(cc546ts); pha-4(zu225) (5x10^{12})	24.306 +/- 0.748	77/90	19.434 +/- 0.441	88/90
smg-1(cc546ts); pha-4(zu225) (5x10^{11})	24.834 +/- 0.682	79/90	20.231 +/- 0.607	87/90
smg-1(cc546ts); pha-4(zu225) (5x10^{10})	27.006 +/- 1.193	50/90	24.957 +/- 0.842	72/90
smg-1(cc546ts); pha-4(zu225) (5x10^{9})	28.521 +/- 0.834	75/90	27.987 +/- 1.213	41/90
smg-1(cc546ts); pha-4(zu225) (5x10^{8})	27.745 +/- 1.073	55/90	19.448 +/- 0.686	57/90
smg-1(cc546ts); pha-4(zu225) (5x10^{7})	18.534 +/- 0.775	61/90	18.068 +/- 0.625	58/90
**Statistical comparison**	**p values**		**p values**	**Combined p values**
smg-1 (5x10^{12})/*smg-1* (5x10^{9})	<0.0001		<0.0001	<0.0001
smg-1; pha-4 (5x10^{12})/*smg-1; pha-4* (5x10^{9})	<0.0001		<0.0001	<0.0001
smg-1 (5x10^{12})/*smg-1; pha-4* (5x10^{12})	0.1674		0.3623	0.2307
Interaction between genotype and food concentration				
Two-way ANOVA *smg-1*/*smg-1; pha-4*	0.3724		0.3927	0.4052
Cox proportional hazard *smg-1/smg-1; pha-4*	0.1537		0.1573	0.1142

B

**RNAi initiated at**			L4			L1		
**Experiment #**			1	2		1	2	
**Strain**	**RNAi**	**Food**	**Mean +/- SD**					
N2	E.V.	AL	20.908 +/- .591	20.683 +/- .611		18.414 +/- .450	18.878 +/- .607	
N2	E.V.	sDR	25.492 +/- .614	27.062 +/-.833		24.812 +/-.629	23.970+/-.616	
N2	*daf-16*	AL	17.876 +/- .359	18.518 +/- .493		16.769 +/- .393	15.512 +/- .325	
N2	*daf-16*	sDR	18.522 +/- .387	19.654 +/- .711		17.938 +/- .423	15.743 +/- .343	
N2	*pha-4*	AL	18.011 +/- .440	19.420 +/- .627		14.422 +/- .304	9.889 +/- .324	
N2	*pha-4*	sDR	20.642 +/- .448	24.539 +/- .650		19.726 +/- .510	12.348 +/- .656	
eat-2	E.V.	AL		22.039 +/- .691		22.127 +/- .580	20.728 +/- .696	
eat-2	*pha-4*	AL		19.881 +/- .619		20.748 +/- .632	12.906 +/- .490	
**Statistical comparison**			**p values**	**p values**	**Combined p values**	**p values**	**p values**	**Combined p values**
N2 E.V. AL/N2 E.V. sDR			<0.0001	<0.0001	<0.0001	<0.0001	<0.0001	<0.0001
N2 E.V. AL/N2 *pha-4* AL			<0.0001	0.1882	0.0002	<0.0001	<0.0001	<0.0001
N2 E.V. AL/N2 *daf-16* AL			<0.0001	0.0031	<0.0001	0.0044	<0.0001	<0.0001
N2 E.V. AL/*eat-2* E.V. AL				0.1199		<0.0001	0.0080	<0.0001
N2 *pha-4* AL/N2 *pha-4* sDR			<0.0001	<0.0001	<0.0001	<0.0001	0.0004	<0.0001
N2 *daf-16* AL/N2 *daf-16* sDR			0.1559	0.1801	0.1284	0.1562	0.4930	0.2744
eat-2 E.V. AL/*eat-2 pha-4* AL				0.0353		0.2460	<0.0001	0.0003

Table S7: sDR increases worm lifespan in a FoxA/*pha-4* independent manner. A) *smg-1* and *smg-1; pha-4* worms were grown at permissive temperature (24° C) until the first day of adulthood when they were switched to 15° C. Experiment #1 is displayed in Figure 4B. B) sDR extends the lifespan of WT(N2) worms treated with empty vector RNAi (E.V.) or *pha-4* RNAi bute not *daf-16* RNAi initiated at larval stage L1 or larval stage L4. L1 Experiment #1 is displayed in Figure S3A. L4 Experiment #2 is displayed in Figure S3B. The mean lifespan values were calculated by a logrank (Mantel-Cox) statistical test from triplicate samples of 30 worms each. n: number of observed dead worms/number of total worms. Combined p values were calculated using Fisher's combined probability test.

sDR-induced longevity is *skn-1* independent	1		2		
**Strain (bacteria conc. /ml)**	**Mean +/- SD**	**n**	**Mean +/- SD**	**n**	
N2 ($5x10^{12}$)			19.387 +/- 0.639	81/90	
N2 ($5x10^{11}$)	20.230 +/- 0.538	59/90	19.069 +/- 0.626	76/90	
N2 ($5x10^{10}$)			21.752 +/- 0.762	69/90	
N2 ($5x10^{9}$)			22.709 +/- 1.015	46/90	
N2 ($5x10^{8}$)	25.448 +/- 0.666	39/90	23.820 +/- 1.023	40/90	
N2 ($5x10^{7}$)			20.450 +/- 0.974	27/90	
aak-2(rr48) ($5x10^{12}$)			17.341 +/- 0.484	80/90	
aak-2(rr48) ($5x10^{11}$)	16.521 +/- 0.447	61/90	17.083 +/- 0.452	70/90	
aak-2(rr48) ($5x10^{10}$)			17.854 +/- 0.602	66/90	
aak-2(rr48) ($5x10^{9}$)			16.622 +/- 0.886	35/90	
aak-2(rr48) ($5x10^{8}$)	16.911 +/- 0.593	43/90	17.869 +/- 0.834	34/90	
aak-2(rr48) ($5x10^{7}$)			16.535 +/- 1.074	29/90	
skn-1(zu135) ($5x10^{12}$)			15.544 +/- 0.358	80/90	
skn-1(zu135) ($5x10^{11}$)	18.547 +/- 0.510	56/90	16.734 +/- 0.449	75/90	
skn-1(zu135) ($5x10^{10}$)			17.220 +/- 0.502	69/90	
skn-1(zu135) ($5x10^{9}$)			19.921 +/- 0.718	50/90	
skn-1(zu135) ($5x10^{8}$)	22.450 +/- 0.575	43/90	20.606 +/- 0.663	58/90	
skn-1(zu135) ($5x10^{7}$)			18.600 +/- 1.199	29/90	
**Statistical comparison**	**p values**		**p values**		**Combined p values**
N2 ($5x10^{11}$)/N2 ($5x10^{8}$)	<0.0001		<0.0001		<0.0001
aak-2 ($5x10^{11}$)/*aak-2* ($5x10^{8}$)	0.4891		0.2566		0.3860
skn-1 ($5x10^{11}$)/*skn-1* ($5x10^{8}$)	<0.0001		<0.0001		<0.0001
N2 ($5x10^{11}$)/*aak-2* ($5x10^{11}$)	<0.0001		0.0049		<0.0001
N2 ($5x10^{11}$)/*skn-1* ($5x10^{11}$)	0.0174		0.0008		0.0002
Interaction between genotype and food concentration					
Two-way ANOVA N2/*aak-2*			<0.0001		
Two-way ANOVA N2/*skn-1*			0.5567		
Cox proportional hazard N2/*aak-2*			0.0205		
Cox proportional hazard N2/*skn-1*			0.7570		

Table S8: sDR increases worm lifespan in an AMPK/*aak-2* dependent, *skn-1* independent manner. Experiment #2 is displayed in Figure 4C. The mean lifespan values were calculated by a logrank (Mantel-Cox) statistical test from triplicate samples of 30 worms each. n: number of observed dead worms/number of total worms. Combined p values were calculated using Fisher's combined probability test.

**sDR-induced longevity is *hsf-1* independent and *clk-1 dependent***	1		2		
**Strain (bacteria conc. /ml)**	**Mean +/- SD**	**n**	**Mean +/- SD**	**n**	
N2 (5x10^{12})	16.695 +/- 0.429	72/90	19.387 +/- 0.639	81/90	
N2 (5x10^{11})	16.714 +/- 0.517	65/90	19.069 +/- 0.626	76/90	
N2 (5x10^{10})	17.772 +/- 0.526	60/90	21.752 +/- 0.762	69/90	
N2 (5x10^{9})	19.627 +/- 0.584	59/90	22.709 +/- 1.015	46/90	
N2 (5x10^{8})	21.482 +/- 0.596	45/90	23.820 +/- 1.023	40/90	
N2 (5x10^{7})	18.269 +/- 0.726	31/90	20.450 +/- 0.974	27/90	
aak-2(ok524) (5x10^{12})	13.976 +/- 0.345	77/90			
aak-2(ok524) (5x10^{11})	13.879 +/- 0.351	64/90			
aak-2(ok524) (5x10^{10})	13.775 +/- 0.310	75/90			
aak-2(ok524) (5x10^{9})	13.897 +/- 0.368	65/90			
aak-2(ok524) (5x10^{8})	13.683 +/- 0.419	58/90			
aak-2(ok524) (5x10^{7})	13.910 +/- 0.397	62/90			
aak-2(rr48) (5x10^{12})	13.786 +/- 0.334	86/90	17.341 +/- 0.484	80/90	
aak-2(rr48) (5x10^{11})	13.584 +/- 0.306	89/90	17.083 +/- 0.452	70/90	
aak-2(rr48) (5x10^{10})	13.871 +/- 0.306	85/90	17.854 +/- 0.602	66/90	
aak-2(rr48) (5x10^{9})	14.125 +/- 0.344	82/90	16.622 +/- 0.886	35/90	
aak-2(rr48) (5x10^{8})	13.723 +/- 0.354	87/90	17.869 +/- 0.834	34/90	
aak-2(rr48) (5x10^{7})	14.604 +/- 0.377	73/90	16.535 +/- 1.074	29/90	
clk-1(e2519) (5x10^{12})	23.536 +/- 0.754	72/90	22.373 +/- 0.765	72/90	
clk-1(e2519) (5x10^{11})	22.638 +/- 0.773	65/90	22.326 +/- 0.823	62/90	
clk-1(e2519) (5x10^{10})	23.387 +/- 0.688	67/90	22.695 +/- 0.788	63/90	
clk-1(e2519) (5x10^{9})	23.120 +/- 0.809	38/90	24.115 +/- 0.905	48/90	
clk-1(e2519) (5x10^{8})	22.627 +/- 0.834	41/90	23.041 +/- 0.853	48/90	
clk-1(e2519) (5x10^{7})	20.100 +/- 1.214	23/90	22.763 +/- 0.928	39/90	
hsf-1(sy441) (5x10^{12})	12.698 +/- 0.330	60/90	12.463 +/- 0.353	68/90	
hsf-1(sy441) (5x10^{11})	12.444 +/- 0.296	60/90	12.075 +/- 0.379	68/90	
hsf-1(sy441) (5x10^{10})	13.201 +/- 0.463	48/90	12.241 +/- 0.404	64/90	
hsf-1(sy441) (5x10^{9})	14.327 +/- 0.440	43/90	14.842 +/- 0.550	23/90	
hsf-1(sy441) (5x10^{8})	14.419 +/- 0.492	36/90	16.125 +/- 0.933	24/90	
hsf-1(sy441) (5x10^{7})	12.517 +/- 0.476	26/90	10.962 +/- 0.684	22/90	
**Statistical comparison**	**p values**		**p values**		**Combined p values**
N2 (5x10^{11})/N2 (5x10^{8})	<0.0001		<0.0001		<0.0001
aak-2(ok524) (5x10^{11})/*aak-2(ok524)* (5x10^{8})	0.7804				
aak-2(rr48) (5x10^{11})/*aak-2(rr48)* (5x10^{8})	0.5787		0.2566		0.4318
clk-1 (5x10^{11})/*clk-1* (5x10^{8})	0.6921		0.6303		0.7982
hsf-1 (5x10^{11})/*hsf-1* (5x10^{8})	0.0003		<0.0001		<0.0001
N2 (5x10^{11})/*aak-2(ok524)* (5x10^{11})	<0.0001				
N2 (5x10^{11})/*aak-2(rr48)* (5x10^{11})	<0.0001		0.0049		<0.0001
N2 (5x10^{11})/*clk-1* (5x10^{11})	<0.0001		0.0014		<0.0001
N2 (5x10^{11})/*hsf-1* (5x10^{11})	<0.0001		<0.0001		<0.0001
Interaction between genotype and food concentration					
Two-way ANOVA N2/*aak-2(ok524)*	<0.0001				
Two-way ANOVA N2/*aak-2(rr48)*	<0.0001		0.0318		<0.0001
Two-way ANOVA N2/*clk-1*	<0.0001		<0.0001		<0.0001
Two-way ANOVA N2/*hsf-1*	0.0253		0.2843		0.0427
Cox proportional hazard N2/*aak-2 (ok524)*	<0.0001				
Cox proportional hazard N2/*aak-2 (rr48)*	0.0012		0.0205		0.0003
Cox proportional hazard N2/*clk-1*	<0.0001		0.0085		<0.0001
Cox proportional hazard N2/*hsf-1*	0.1290		0.6568		0.2938

Table S9: sDR increases worm lifespan in an AMPK/*aak-2* and *clk-1* dependent, *hsf-1* independent manner. Experiment #2 is displayed in Figure 4C. Experiment #1 is displayed in Figure 5. The mean lifespan values were calculated by a logrank (Mantel-Cox) statistical test from triplicate samples of 30 worms each. n: number of observed dead worms/number of total worms. Combined p values were calculated using Fisher's combined probability test. Note that experiment #2 of Table S9 was performed at the same time as experiment #2 of Table S8.
